# Design and evaluation of a collaborative UML modeling environment in virtual reality

**DOI:** 10.1007/s10270-022-01065-2

**Published:** 2022-11-19

**Authors:** Enes Yigitbas, Simon Gorissen, Nils Weidmann, Gregor Engels

**Affiliations:** Paderborn University, Zukunftsmeile 2, 33102 Paderborn, Germany

**Keywords:** Collaborative modeling, Virtual reality, UML

## Abstract

Modeling is a key activity in conceptual design and system design. Through collaborative modeling, end-users, stakeholders, experts, and entrepreneurs are able to create a shared understanding of a system representation. While the Unified Modeling Language (UML) is one of the major conceptual modeling languages in object-oriented software engineering, more and more concerns arise from the modeling quality of UML and its tool-support. Among them, the limitation of the two-dimensional presentation of its notations and lack of natural collaborative modeling tools are reported to be significant. In this paper, we explore the potential of using virtual reality (VR) technology for collaborative UML software design by comparing it with classical collaborative software design using conventional devices (desktop PC/laptop). For this purpose, we have developed a VR modeling environment that offers a natural collaborative modeling experience for UML Class Diagrams. Based on a user study with 24 participants, we have compared collaborative VR modeling with conventional modeling with regard to efficiency, effectiveness, and user satisfaction. Results show that the use of VR has some disadvantages concerning efficiency and effectiveness, but the user’s fun, the feeling of being in the same room with a remote collaborator, and the naturalness of collaboration were increased.

## Introduction

In modern software development, collaboration between developers is one of the driving factors that determines the quality and speed at which projects can be realized. One central artifact of communication and discussion in software engineering are models [50]. The Unified Modeling Language (UML) with its associated diagrams is one of the most well-known general-purpose modeling languages in software engineering and is considered by many as “lingua franca” for software engineers [35]. However, researchers and software designers have realized the insufficiency of UML in its expressiveness. The expressiveness of a conceptual model depends on the set of language symbols used for representation. Since UML is restricted to a two-dimensional plane, these insufficiencies concerning expressiveness include a lack of dynamic expression and interaction ability between groups of remote designers  [18, 32] as well as the complexity for large models [13]. Furthermore, the authors in [6] argue that the dissatisfaction of developers with UML tools is one of the reasons it has not been adopted more universally, exemplifying the need for improving the tool support. Since the COVID-19 pandemic has spread around the world, this need for good UML tool support has only increased. Many educational institutions, like schools and universities, around the world, have been forced to switch to online education settings to support social distancing. Likewise, millions of workplaces wherever possible were transitioned to home office. To enable collaboration for software engineers in such situations, tools are needed that offer support for creating and discussing models from remote locations. While classical modeling applications, like *Lucidchart* [26] or *GenMyModel* [2], support remote collaboration, they do not overcome the mentioned issues with regard to visualization and collaboration as they are mostly relying on a 2D UML notation and do not support a natural way of collaboration comparable to editing a model on a whiteboard while situated together in one room.

Virtual reality technology, on the other hand, is becoming increasingly sophisticated and cost-effective and can be applied to many areas such as training [56], robotics [58], education [61], finance [52], and even Information Systems (IS) research [51], to simulate a real environment or represent complicated scenarios. Modern head-mounted display (HMD) virtual reality (VR) devices have several technological capabilities that are not present on conventional devices (desktop PC/laptop): (1) stereoscopic 3D images, (2) six degrees of freedom, (3) hand presence support, and (4) 3D spatial voice chat. Since HMD devices show a slightly different image to each eye, they can invoke the perception of a truly 3D virtual world that the user inhabits. These stereoscopic 3D images are more in line with the visual experience of the real world compared to images on a 2D screen, since people also perceive the real world in 3D, not in 2D. In VR, users can also look around in the virtual world by simply moving their heads, like they would in a real environment. In addition to this rotational movement, modern VR devices can also track the positional movement of a user, independent of the direction the user moves in. This means the user has Six Degrees of Freedom (three rotational and three positional axes) in her/his movement and the VR system can adapt the view and position of the user in the virtual world accordingly. Additionally, typical VR controllers are used single-handedly with each controller representing one hand in the virtual world. This hand presence support allows a user to make natural gestures like grabbing an object and pointing at things to interact with the virtual world. With the means of 3D spatial voice chat, it is possible to make users feel like the voices of their collaborators come with the volume level (distance-based) and from the direction that their virtual representations (i.e., Avatars) are in. Furthermore, gamification is an essential concept that is highly associated with VR technology. The goal of gamification is to create a gameful experience for the users of a service in order to improve motivation to engage with an application or task [21].

Overall, the fast development in VR concerning prototyping [23] and engineering of VR applications [57] as well as the mentioned technological advances allow us to extend the research field to improve the quality of UML and collaborative modeling. Thus, the main goal of this paper is to explore the potential of using VR technology for collaborative UML software design by comparing it with classical collaborative software design using conventional devices (desktop PC/laptop). For this purpose, in our previous work [53], we have developed a VR modeling environment, called *VmodlR*, that offers a natural collaborative modeling experience for UML Class Diagrams. In the present work, we build up on the *VmodlR* solution idea and extend it by several aspects. First of all, we have updated our literature survey on collaborative modeling tools and present a more detailed analysis of related approaches in this field. Furthermore, we elaborate on the conceptual solution by covering several aspects such as model synchronization, visualization, and editing in VR. Similarly, the technical details for implementing our collaborative UML modeling environment in VR are presented in more depth compared to our original work. Finally, we present the evaluation of our work based on a user study. Here, we have compared collaborative VR modeling with conventional modeling with regard to efficiency, effectiveness, and user satisfaction. In addition, we present further results about motivational aspects of VR for modeling purposes. The main results of our usability evaluation show that the use of VR has some disadvantages concerning efficiency and effectiveness, but the user’s fun and motivation, the feeling of being in the same room with a remote collaborator, and the naturalness of collaboration were increased.

The rest of the paper is structured as follows. In Sect. 2, we present and discuss the related work. In Sect. 3, we describe the conceptual solution of our VR-based collaborative modeling environment *VmodlR*. In Sect. 4, we show the details of the implementation of *VmodlR*. In Sect. 5, we present and discuss the main results of the usability evaluation. In Sect. 6, we conclude the paper and give an outlook for future work.

## Related work

Model-based and model-driven development methods have been discussed in the past for various application domains [5]. In the following, we draw on prior research into *collaborative modeling*, *3D modeling*, and *immersive modeling*.

### Collaborative modeling

In previous work, the topic of collaborative software modeling was already researched from different perspectives [40]. In [8], the authors present a distributed UML editor that aims to transfer collaborative discussion and editing of UML models from regular meeting rooms to remote settings using regular computers. Recker et al. [41] proposed a system based on the Second Life platform^1^ where users can collaboratively create process models while being represented by an avatar with the ability to voice chat. The models are represented on a tile-based 2D plane in a 3D world on which the avatars can walk around. The application is created with desktop systems in mind. They evaluate their work with a small user study to gain feedback from users that collaboratively created a process model. Results indicate that the users found the interaction to be very natural and the collaboration to work very well in the virtual world, demonstrating the benefit of using a 3D world and virtual avatars to represent users. In addition, a collaborative learning environment for UML modeling, called CoLeMo, is presented in [11]. CoLeMo is designed for students studying UML modeling. It can also be used as a platform for collaborative design of software. Furthermore, [15] introduces an approach to a real-time synchronous collaborative modeling of software systems using 3D UML.

Besides these approaches from research, there are also some commercial tools supporting collaborative software modeling. *Lucidchart* is a web-based commercial tool for collaboratively creating diagrams [26]. It includes support for many diagram types and modeling languages like UML, Business Process Model Notation (BPMN), Enterprise Relationship models, and more. The tool shows a 2D canvas where standard 2D diagrams can be created. Since Lucidchart is web-based, it can be used by any device that runs a modern browser, although as with many web-based contents the experience can be assumed to be best suited to the use on desktop and laptop computers and is not specifically adopted to the possibilities of VR devices. Released by Axellience in 2014, GenMyModel [2] is a web-based tool similar to Lucidchart. GenMyModel supports a subset of the most commonly used UML diagrams, i.e., Class, Use Case, Component, Object, State, Deployment, Activity, and Sequence Diagrams. Axellience describes the mode of collaboration as similar to how Google Drive or Microsoft Office online collaboration works. So users can see where their co-workers are editing the model but do not have an integrated voice chat to discuss those changes. This has to be done via an external tool. The system aims to support the language’s visual representations according to their official definitions and thus only presents 2D models to the users.

While the above-described approaches mostly support collaborative modeling based on standard 2D UML diagrams, they do not support immersive VR. Immersive VR, however, is essential for enabling a natural and more interactive way of remote collaboration in modeling.

### 3D modeling

While most of the existing UML modeling tools rely on a 2D representation, there are also ones covering a 3D model representation and UML visualization. The main goal of these approaches, which will be described in the following, is to discover whether using a 3D perspective has a substantial impact on model comprehension.

The authors in [38] presented a conceptual system that visualizes UML Class and Sequence Diagrams in 3D. The Sequence Diagrams are displayed in the context of the Classes they belong to and animated to emphasize their connection. Furthermore, the authors in [63] have also discussed a conceptual approach to extending 2D UML Diagrams to the third dimension. They introduce some modeling quality attributes and explain how VR can improve the UML modeling quality according to these attributes. For example, they argue that the model’s “understandability” can be improved through VR’s “Immersion” and “Stereopsis” (stereoscopic 3D presentation) features. Subsequently, they provide some examples of a Class Diagram and a Sequence Diagram that are visualized in 3D and demonstrate their advantages in comparison with a 2D representation. Herpich et al. [22] presented *VirtualLab*, a system focused on offering a new learning environment for teaching software engineering, specifically UML, in a 3D virtual environment. Modeling is possible through a number of panels embedded into the virtual environment. Each panel shows one web instance of the GenMyModel modeling tool (cf. Sect. 2.1). Their evaluation focuses on the learning effectiveness of their prototype. The underlying technology they use for the virtual world is Open Simulator.

In [42], the authors proposed VisAr3D, a 3D visualization tool for UML models to make large models easier to comprehend, especially by inexperienced modelers. The system automatically converts a 2D UML model from an .xmi file into its 3D representation. The virtual environment, the 3D UML models are placed in, can be viewed through a web-based app and does not support HMDs. Their prototype is not a model editor, however, it only visualizes UML models in 3D. This approach was evaluated based on a user study assessing the effect of the 3D compared to a 2D representation. The main results show that the 3D representation aided the understandability of large models, “increased students’ interest” and supported teaching purposes [42]. In [25], the authors described an implementation that visualized a process model in a 3D virtual world on a desktop computer. It is a training system intended to aid a single user in understanding Business Process Models through 3D visualization. They evaluate how the 3D representation assists the user in learning the modeled process compared to a standard 2D model representation. They conclude that the 3D representation provides a noticeable learning benefit. It is not a modeling tool, however, only a visualization and training tool. Further similar examples for approaches that make use of a 3D representation of UML models can be found in [10, 27, 48, 49]. In summary, a modeling approach that combines and integrates the aspects of 3D modeling, collaboration, and VR in one solution is not fully covered and yet existing.

### Immersive modeling in AR and VR

In the following, augmented reality (AR)- and virtual reality (VR)-based approaches for modeling purposes will be briefly described and discussed. Although VR is our main focus, we included AR approaches to cover immersive modeling approaches on the whole.

AR is closely related to VR with the main difference being that VR immerses a user in a completely virtual world while Augmented Reality (AR) does not isolate the user from the real world by displaying virtual objects in the real environment. In general, AR has been already applied for different aspects such as robot programming [54], product configuration (e.g., [19, 20]), planning and measurements [60] or for realizing smart interfaces (e.g., [24, 55]). To be more specific, example approaches that apply AR for software modeling are as follows. The authors in [28] have presented a framework that is supposed to allow editing and viewing UML models in a 3D space through the Microsoft Hololens AR Glasses. Their approach is to overlay 3D model elements over real scenery so the user can move around and inside the model while not being shut off from the real world. In this way, their prototype only displays static objects that are meant to represent UML models. Similarly, in [39], the authors proposed a system that aims to make learning UML more accessible by displaying it in 3D as overlays to the natural environment using the Microsoft Hololens. The system supports creating and editing UML Class Diagrams but is only intended for single users and does not support collaboration. Furthermore, in [44] the authors introduce the concept of “HoloFlows” to support the modeling of processes for the internet of things in mixed reality. A similar solution is introduced in [4] where the authors present an approach for supporting domain-specific modeling environments based on AR. The main drawback of AR-based solutions for modeling is the small field of view which narrows down the possibilities for modeling support. Therefore, we have explored an alternative solution in VR.

Focusing on VR-related approaches for software modeling, we can see that many previous works already have seen the potential in using VR for improving modeling activities. In [33], for example, the authors have presented a system that analyzes an object-orientated code base and visualizes its classes, attributes, and relations automatically in a 3D virtual environment that users can inspect inside a Cave Automatic Virtual Environment (CAVE). So it supports VR only in a broader sense. The application is not networked and does not support editing the model, it is only a visualization tool. In [12], the author has proposed a system for HMD VR devices that can import a Finite State Machine (FSM) and visualize it in a game-like environment where players stand on islands representing states and can change islands via different boats representing the possible transitions. It is a single-player game environment meant for educational purposes and does not support UML modeling with actual UML Diagram elements or remote collaboration. In [34], the authors have presented an HMD VR system for visualizing process models in 3D. Therein, the models can be annotated but not edited. They evaluated the effectiveness, efficiency, and intuitiveness of the VR visualization in comparison with (1) paper and (2) desktop tool-based BPMN and found that the effectiveness was equivalent between VR and desktop, but task completion in VR was faster than with the BPMN tool and the users found the VR controls very intuitive. Zenner et al. [62] proposed a system that visualizes an event-driven process chain (EPC) model in 3D for a user to explore using an HMD VR system and real-world haptics. The approach is focused on aiding the learning process and is intended for single users. In a user study focusing on model understandability and user experience comparing the HMD VR experience with viewing a 2D process model, they yielded that users understand the model faster in 2D while “the users’ interest” is higher in VR. Pöhler et al. [37] implemented a VR-based process model editor where users can collaboratively create business process models. It supports HMD and desktop systems with native voice-chat. The modeling language used was a scaled-down version of BPMN that was customized for ease of use and intuitiveness. They evaluated the system with a small user study focused on usability with users stating the modeling in 3D was especially helpful since the modeled processes were semantically linked to virtual objects in the scene.

In summary, existing immersive modeling tools do not support remote collaborative modeling where UML models can be created and edited with multiple stakeholders in a shared virtual environment.

### Discussion

Table 1 shows the combined overview of the previously discussed contributions in regard to what features their prototypes support and what features are only conceptually discussed.

**Table 1:**
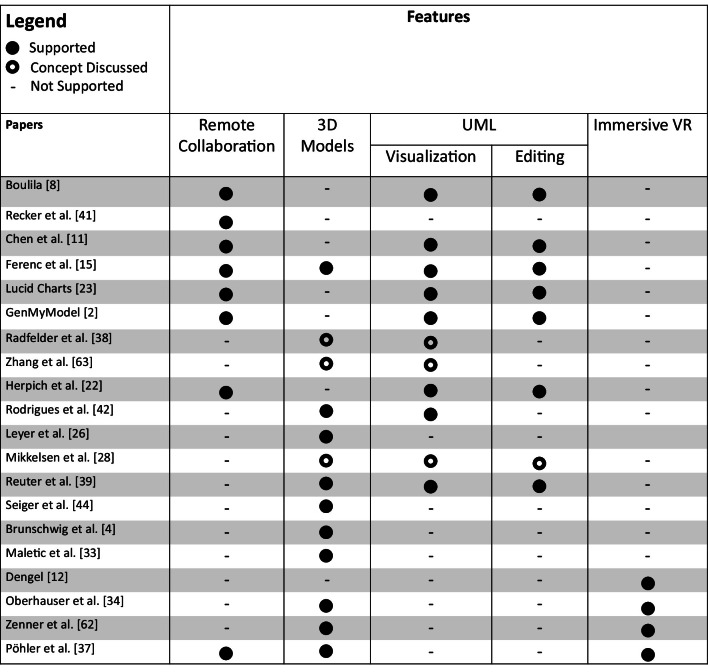
Overview and comparison of related approaches

This table shows that, in the area of AR and VR modeling, 3D models are examined quite often. This is expected as a true 3D representation is one of the technological benefits of many VR and AR devices compared to regular computer screens. Additionally, the table exemplifies that while UML is covered rather often in work targeting desktop systems and conceptual discussions on 3D UML representations have been published in the past, there are still only a few contributions using UML in a VR or AR context. Here lies an open research gap that still needs to be filled.

**Fig. 1:**
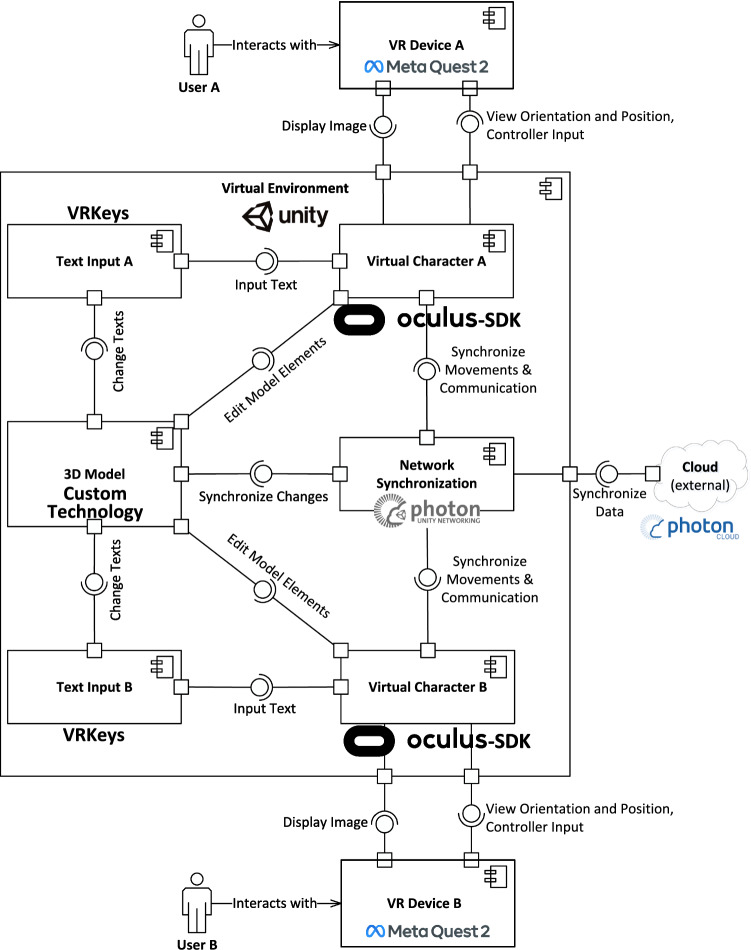
System overview of *VmodlR*

While UML has been given little attention in AR and VR, there are some contributions in this field covering other modeling languages like BPMN and EPC. They usually also include a translation from the two-dimensional formal definitions to a 3D representation of the models and indicate that this offers tangible benefits to aspects like understandability and motivation [39, 42]. Although this is still in need of scientific proof, it can be expected that these benefits are independent of the modeling language and will transfer to 3D UML models in VR.

Collaboration seems to be very prevalent on desktop computers with very little emphasis on it in VR and AR applications. This is possibly due to AR and VR devices in their modern form being relatively new combined with applications being generally more complex to develop if they support collaboration compared to only supporting single users. The only collaborative modeling environment in VR or AR we found was Pöhler et al.’s system [37] which does not support UML and only uses 3D structures of 2D objects instead of fully 3D model elements.

Therefore, our literature review has shown that the four core features that identify a collaborative UML modeling environment in VR have not been implemented in an application together before. However, all of them are present individually in different works about AR, VR, or desktop prototypes. This means there are multiple sources an implementation of a collaborative UML modeling environment in VR can draw from to make informed design decisions, while this new implementation still comprises a novel approach. Furthermore, our literature review indicates that no study so far has focused on a comparison between VR and desktops in regard to the naturalness of collaboration in the context of modeling yet. Therefore, the results of this review imply that the approach we have taken in this work is a novel contribution, both in regard to the implemented solution and its evaluation.

## Solution overview

The system overview of our VR-based collaborative modeling environment *VmodlR* is shown in Fig. 1. The top half of this figure represents *User A* and the *Virtual Environment* this user accesses through a *VR Device*. The bottom half symmetrically shows a remote collaborator, *User B*, and the *VR Device* this user employs to access the same shared *Virtual Environment*. Through the *VR Device*, each *User* is represented in the *Virtual Environment* as a *Virtual Character*. The *View Orientation and Position* of the *VR Device* together with the *Controller Input* controls the *Virtual Character* while the *Virtual Environment* with its content is displayed from the *Virtual Character*’s perspective inside the *VR Device*.

Through the *Virtual Character*, each *User* interacts with the elements inside the *Virtual Environment*: The *3D Model* can be edited by either directly editing model elements (e.g., creating, deleting, or moving them) or through the *VR Text Input* component that is used to edit the text inside the *3D Model*, for example, the Class names. All those changes to the model are synchronized between users through the *Network Synchronization* component. In the case of this solution, the *3D Model* is a three-dimensional UML Class Diagram, but theoretically, this could be adapted to any kind of conceptual model. Through their *Virtual Characters*, *User*s can also interact with each other via *Network Synchronization* which transmits their voices and synchronizes their body and hand movements. This is visualized in Fig. 1 as the *Synchronize Movements & Communication* interface between the *Virtual Character*s and the *Network Synchronization*. The *Network Synchronization* component then ensures that the *Virtual Environment* and its content are synchronized between the *User*s through the *Cloud*. It offers several different services that can be used by other components to synchronize all necessary aspects of the *Virtual Environment*. In the following, each of the main components *Virtual Environment*, *Network Synchronization*, *Virtual Character*, *3D Models*, and *VR Text Input* will be described in more detail.

### Virtual environment

The *Virtual Environment* consists of all virtual elements that are needed to provide a collaborative software modeling environment. Within this environment, each user can see and move around via a *VR Device* in 3D. As a design decision for the *Virtual Environment*, we opted to use an open space instead of a closed one to not introduce some unrealistic environment behavior or limit the user’s ability to create large models. Since there is no open environment that could be considered natural for creating conceptual models, any space that offers a planar ground for the user to walk on could be chosen. We decided on a grass field under a blue sky because it does not limit the 3D space available to the user and depicts a pleasant real-world environment that users are familiar with.

**Fig. 2:**
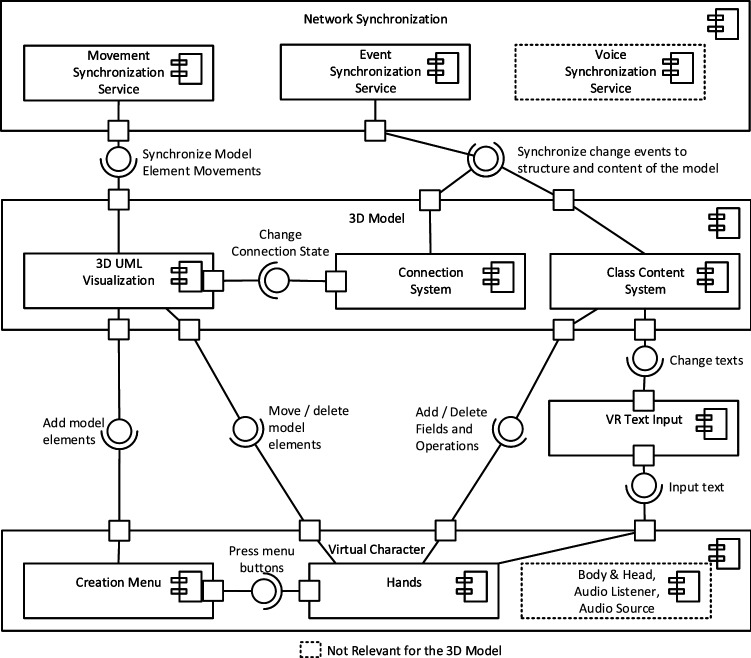
Detailed view of the *3D Model* from Fig. 1 and its relations to the *Virtual Character*, *Network Synchronization*, and the *VR Text Input*

### Network synchronization

All elements inside the *Virtual Environment* have to be synchronized through the *Network Synchronization* component. We will describe how this networking generally works in this conceptual solution. The networking has a server-based architecture where all users connect to a server and the server synchronizes instances of a *Virtual Environment* between all users that are currently in that environment. It is important to understand that a networked environment is not a singular environment. Instead, on each user’s *VR Device* (clients) a local version of the networked environment exists, so the user can look around in it and interact with it. All the changes the user can thereby make inside the *Virtual Environment*, like moving their *Virtual Character*, are communicated to the server so it can update its reference representation of the networked environment and forward the changes to the local environments of all other clients. These clients then apply the changes accordingly to their local copies of the environment. This way, the local environments on all clients are always kept in sync with the server’s networked environment. As shown on the top of Fig. 2, three components can be used to synchronize objects inside the *Virtual Environment*: The *Movement Synchronization Service*, the *Event Synchronization Service*, and the *Voice Synchronization Service*.

The *Movement*- and *Event Synchronization Service*s can be used in a more general way. The difference between them is that the *Movement Synchronization Service* is dedicated to rapidly and frequently changing information, that needs to be synchronized many times per second. In the case of movement, this is needed to show the movement of an object that is moved by a remote collaborator fluently to the local user. The *Movement Synchronization Service* also deals with tracking if a synchronized object that can be moved gets created or deleted. The *Event Synchronization Service*, on the other hand, is supposed to synchronize arbitrary events that happen rather infrequently and therefore only have to be synchronized occasionally, once they occur, instead of the constant synchronization needed for movement. This provides flexibility where something that only occasionally changes (like the name of a Class for example) can be synchronized via the *Event Synchronization Service* and otherwise does not consume network bandwidth while things that often change and have to be synchronized many times per second, like the movement of a Class or a *Virtual Character*’s *Virtual Hands*, can be synchronized fluently via the *Movement Synchronization Service*. Finally, the *Voice Synchronization Service* is dedicated to synchronizing the voices of users to enable voice chat inside our solution.

### Virtual character

In VR, people can be represented by 3D characters through an avatar with a body, head, and hands. This way, a user can, for example, move around in the virtual environment and point with her/his finger in real life and the *VR Controller* can reproduce this gesture on the *Virtual Character*’s hands. Since this has the possibility to make discussions about models in VR much more natural than possible on PCs, these features were also included in the design of our solution. The hands’ gestures are synchronized across the network for each user, along with the positional audio of voices and the positions and orientations of *Virtual Character*s. Our solution supports this form of natural movement, where the *Virtual Character* and therefore the user’s view of the world changes according to the physical *VR Headset* movement. This is the ideal scenario for movement, where there is enough physical space available to the user to move anywhere the user would want to go in the virtual world. However, this is hardly a realistic scenario since the models creatable in our solution can theoretically become arbitrarily large, meaning that the user would need an infinitely large physical space to move around in. Therefore, a secondary movement method, namely teleportation is required, that allows a user to move their *Virtual Character* through the environment without moving in the physical world. Teleportation involves the user entering a teleportation mode, for example through pressing or holding a specific button on one of the *VR Controllers* and then aiming the controller at a spot that the user wishes to teleport to. When either releasing the button or pressing it again the target position is selected and the *Virtual Character* is instantly teleported to the aimed location. With these two movement methods—natural walking and teleportation movement—the user can reach any position in the *Virtual Environment* independent of the size of physical space available to the user while still moving in a rather natural way.

### 3D models

To take full advantage of the stereoscopic 3D images that *VR Device*s can present to the user, the UML Class Diagrams this work focuses on, need to be represented in 3D as well. Additionally, the user needs a way to edit this 3D model, for example, by changing the text on Classes, creating new model elements, or deleting old ones. These aspects will be described in the following. Figure 2 shows a detailed view of how the *3D Model* from Fig. 1 is set up and with which parts of the *Virtual Character* and the *Network Synchronization* components it interacts. Note that those parts of the *Virtual Character* and *Network Synchronization* not relevant for the *3D Model* are occluded here for a better overview.

In the following, first, a description of the design of the *3D UML Visualization* without its interactions with other components will be provided. All of the interactions and other components are changes to the model that can be made by the user through the *Virtual Character* which are then synchronized to the other clients through the *Network Synchronization*. This editing of the *3D Model* will be discussed afterward.

#### 3D UML visualization

Usually, modeling languages are only specified with 2D visual representations. UML is no exception to that rule. We could stick to those same 2D shapes inside a 3D world with users being able to position the 2D shapes freely in 3D. However, we believe using 3D shapes instead of 2D ones will likely result in a more natural experience for users because the real world only consists of 3D objects and we do not want the model elements to seem like foreign bodies in the *Virtual Environment*. The main shapes used to visualize UML elements in the 2D specification are rectangles and lines with different forms of arrowheads at the end of those lines. To ensure that users familiar with the 2D representations can learn the 3D ones easily, we tried to find natural equivalents of the 2D shapes in 3D. The equivalent of rectangles in 3D is cubes and cuboids, while lines are best represented by thin tubes (see Fig. 8c). A challenge of a 3D visualization of UML Classes is the text representation inside them. A 2D Class only has one side that the text is displayed on which always faces the user. Since the Class rectangles known from 2D are most similar to Cuboids in 3D, every UML Class is visualized as a cuboid in our solution (see Fig. 8a). It can be assumed that the users can see at most three of the six sides of a cuboid at any given time. If the Class’s cuboid only displays the text on one side, this side could, thus, be hidden depending on the view direction that the user has toward the Class. Rotating the cubes automatically so the text side always faces the user would be a possible solution to that. However, we wanted the user to be the only entity changing the model’s appearance, so the model seems more stable and thus natural. For these reasons, we display the text associated with a Class on all sides of the Class. This means that every Class side basically shows the same 2D Class in a notation similar to 2D UML. Since all these sides belong to a single Class, it is important that all sides always show the same content.

#### Model editing

A central aspect of any modeling tool is the control regarding how to create and manipulate the actual models. This subsection will discuss how a user can create and manipulate a *3D Model* through the system visualized in Fig. 2. First, we will cover how a *Virtual Character* can create and delete model elements from the *3D UML Visualization*. Subsequently, we will cover how these elements can be moved and how, based on that movement, Connections can be attached to and detached from Classes via the *Connection System*. Lastly, it is explained how the content of a Class can be edited through the *Class Content System*. Note that the internals of the *VR Text Input* component will be covered at the end of this section.

***Creating and Deleting Elements:*** To create new model elements inside the *3D Model*, some form of Graphical User Interface (GUI) (i.e., menu) is needed. Such a *Creation Menu* is shown in Fig. 2 in the bottom left corner. To ensure consistency within the solution, the general control concept used for the *Creation Menu* should also be used for other GUIs inside the application.

There are two general possibilities when it comes to controlling menus in VR: Laser pointer controls and intersection controls. The system menus of the Meta Quest 2^2^ and Meta Rift S^3^ VR devices are examples of laser pointer-based menus: The user has a virtual laser pointer attached to one of her/his hands which works as a mouse pointer and uses a button to select interface elements that the laser pointer points at. Intersection controls aim to make the interaction more natural than laser pointing at elements, by having the user push buttons and select elements by moving the controller, and thereby the virtual hand, into a button to push it down, similar to how a real button would work. Therefore, one main difference between these methods is that intersection-based methods require more movement since the user has to physically reach the elements while laser pointer controls are usable from a distance. The decision between the two is therefore a trade-off between an option that can be assumed to be more natural to users and an option that may be more convenient and versatile. Since this solution is supposed to offer only one method of interacting with GUI elements for consistency reasons, we chose laser pointer controls for interacting with menus. The laser pointer can be switched on or off, so it does not distract from the modeling interactions while the user is not controlling GUIs.

The second part of the GUI design, besides the controls, is the visual representation. In many applications, and also, for example, in the Meta Quest’s system menus, the VR GUIs are 2D panels, like screens, placed in the virtual environment. An alternative to that would be representing UI elements as virtual three-dimensional objects like discussed by Greenwald et al. [16], to give the interface elements more plasticity and make them closer to real objects. This is especially sensible when the interface elements represent real-life objects, like Greenwald et al.’s painter’s palette. For some GUI elements in this solution, such a natural equivalent is not obvious or does not exist. For others, using them could be distracting. For example, buttons to create new model elements could be small versions of these elements. This has the problem that it could lead to confusion about if those elements are already created objects or just the creation buttons. This is why we decided to stick to 2D representations for all interface elements which also ensures consistency within the application, as discussed before. This way, especially the creation buttons cannot be confused for parts of the model or the environment. Similar to VR apps like AltspaceVR [31] and Spatial [45], the *Creation Menu* sticks to the user’s *Virtual Character* and follows the horizontal view direction of the user so it is always easy to find. The menu is positioned to the bottom of the user’s *Virtual Character* so it does not occlude the user’s vision when looking straight ahead or up during regular modeling. It is also tilted to face the user for easier reading. This form of the menu will be familiar to regular VR users while those unfamiliar with VR do not have to get used to a different menu representation or control scheme between the system menus of many VR devices and the in-application menus making it easier to use for both seasoned VR users and novices alike.

Object deletion works very similarly. The main difference is that there is no dedicated *Delete* button on each model element and no deletion menu analogous to the *Creation Menu*. Instead, the user can activate a deletion mode via a controller button. In this mode, the laser pointer can be used to select a model element and delete it by pressing the enter button also used for GUIs. This system makes sure that creating an object by pointing the hand at a create button and pressing the enter button works similarly to deleting. This should make it rather easy to learn and remember the concepts of how model elements are created and deleted.

All of the changes to the *3D UML Visualization* through creation and deletion of model elements are synchronized across the network through the *Movement Synchronization Service* (cf. Fig. 2). This is done because all of these model elements can also be moved and this way the *Movement Synchronization Service* can handle the entire life cycle of a virtual object from being created, via being moved around, to being ultimately deleted. How this movement works in detail will be discussed in the following.

***Moving Model Elements:*** One central interaction in any modeling application is moving model elements. A UML tool is no exception: Classes have to be moved around and Connections, e.g., Associations, have to be moved and attached to the different Classes. In a VR application, especially with our focus on natural interactions, there is one obvious way to design this feature: Grabbing. In Fig. 2, this is visualized as the *Move model elements* interface between the *Virtual Character*’s *Hands* and the *3D UML Visualization*.

In many VR applications, objects can be grabbed with grab buttons that exist on many VR controllers. These buttons are explicitly designed to simulate the real movement of the fingers when grabbing an object by being positioned under the left and right middle fingers, respectively. The movement of the middle finger toward the palm of the hand simulates the hand closing when grabbing an object and releasing it will let loose that object. To emphasize the notion that users are actually grabbing and holding objects, we did not include a feature to grab objects from a distance in our design.

For Classes, this grabbing interaction is therefore very straightforward: A user intersects her/his *VirtualCharacter*’s *Hand(s)* with the Class and presses and holds the grab button to move it around. While the button is pressed, the Class sticks to the *Hand* and once it is released, the Class stays in its new position. During that process, the Class behaves like a real object would, meaning it can be turned around and moved in whatever way the user wants. This is supposed to make the object’s behavior easy to understand and predictable due to its naturalness. Of course, a notable exception to that is the lack of gravity since with it, objects would just fall to the ground and it would be impossible to create truly 3D models.

The second main type of model elements supported are Connections which, in their most basic form, consist of a straight line possibly with one of several arrow-shapes at one end. In many UML modeling and diagramming tools, like Microsoft Visio [30], or Lucidchart [26], each end of a connection can be attached to a Class and will stick to that Class when the Class is moved. They are typically visualized as lines connecting visual dots. These dots can be moved and attached to elements like Classes. For non-angled Connections that only consist of one straight line, only the start and the end of the line need such moveable points. To provide a level of familiarity between the 2D tools and our 3D solution, it is sensible to use a similar mechanism for moving and attaching connections.

The movement and attaching of connections in a 2D program is usually handled by moving the dots that belong to a connection with a mouse. The natural equivalent of dragging and dropping elements with a mouse is grabbing and moving them in VR with the VR controllers since these are the only pointing instruments a VR user has. Using the grab buttons on the controllers for this “grabbing”-action ensures consistency between the control methods used for moving Classes and Connections. Thus, users are able to grab and move the start and end points of a Connection with their *Hands* like they can grab a Class.

Besides moving a Connection, users also have to be able to attach each of its ends to a Class, so that when the Class moves, the Connection sticks to it. This is done by releasing the respective end while that end points toward the Class it should attach to. The Connection automatically searches, along the direction of its line, for a Class that it can attach the previously grabbed end to. This makes sure the attaching process is easy to perform since it is directly linked to moving the connection and does not require additional interactions.

**Fig. 3:**
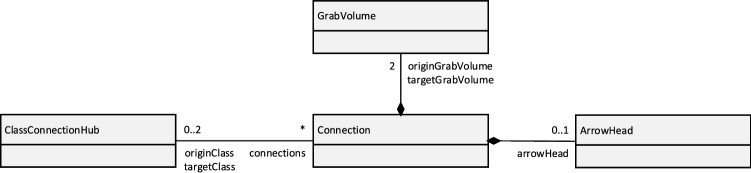
Internal structure of the connection system

The *Connection System* shown in Fig. 2 tracks what connections are connected to which Classes. Therefore, when a Class inside the *3D UML Visualization* is moved, the *Connection System* can instruct the connections attached to this Class to update their positions, so it appears as they stick to the Class while it is being moved. The internal structure of the Connection System is shown in Fig. 3.

The *ClassConnectionHub* is a part of every Class and tracks which *Connection*s are attached to it. A *Connection* consists of one *GrabVolume* at each of its ends and an *ArrowHead* which is the arrow shape at the target end of the *Connection*. The *GrabVolume*s are the points of the *Connection* that can be grabbed and moved.

Each *Connection* from the *Connection System* is bound to a specific visual Connection from the *3D UML Visualization* providing that visual element with the respective functionality. Likewise, each *ClassConnectionHub* is also bound to the visual representation of one specific Class. The *Connection System* therefore closely works together with the *3D UML Visualization*.

**Fig. 4:**
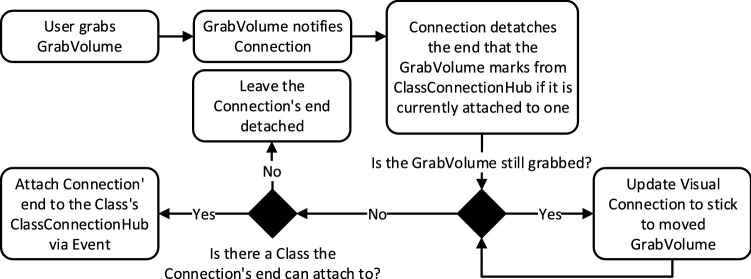
Procedure of moving a *Connection*’s end and attaching it to a class

Figure 4 shows how this structure is used to move a Connection’s end and attach it to a Class.

When one of the *GrabVolume*s is grabbed, it notifies the *Connection* which detaches the respective Connection end from the Class it is currently attached to. It does so by updating its internal attachment state and notifying the *ClassConnectionHub* that its grabbed end is currently attached to if there is one. This triggers a change event which is synchronized via the *Event Synchronization Service* (See Fig. 2).

As long as the *GrabVolume* is moved, the position, length, and rotation of the *Connection*’s visual representation are updated to always lead from the grabbed *GrabVolume* to the other one. This transformation information is synchronized via the *Movement Synchronization Service* of the Connection’s visual elements from the *3D UML Visualization*.

When the *GrabVolume* is released again, the *Connection* is notified by the *GrabVolume* that this loose end of the *Connection* should try to attach to a Class again. The *Connection* then searches along the direction of the current Connection line toward the loose end for a *ClassConnectionHub*.

However, it specifically does not stop searching when the line ends but goes further than that, so it finds a Class in that direction if there is one, independent of if the *Connection*’s line already touches it or not. When such a *ClassConnectionHub* is found, the *Connection* attaches to the Class via another networked event. The respective *Connection* catches this event on every client and attaches itself accordingly by updating its internal connection state and notifying the *ClassConnectionHub* that the *Connection* just attached to it. However, if no *ClassConnectionHub* is found, no network event is sent and the *Connection*’s end stays loose.

This way, a *Connection* always knows to what *ClassConnectionHub*—and thereby to which Class—each of its ends is attached and the *Connection* can be moved and attached to different Classes through grabbing and moving the *GrabVolume*s.

Additionally, each *ClassConnectionHub* knows which *Connection*s are attached to it. When a Class is grabbed and moved, its *ClassConnectionHub* notifies the *Connection* to update their visual element, so the *Connection* stays fixed to a specific point relative to the Class. That means that the line’s orientation and length as well as the position of the *ArrowHead* are updated to always connect the same two points on the two attached Classes even while one or both of them are being moved. This is synchronized across the network through the *Movement Synchronization*, just like when a *Connection*’s end is moved via a *GrabVolume*.

***Class Content System:*** Besides setting up the structure of Classes and their Connections to each other, another major part of UML Class Diagrams are the contents of the Classes themselves. This includes their names, the list of fields, i.e., attributes, and their list of operations, i.e., methods. The basic visual setup of Classes, consisting of six mirrored sides on a cuboid that shows the Class in a similar way to common 2D representations, was already discussed before. The following two subsections focus on how users can interact with them and how changes on one side of a Class are synchronized to the other sides and across the network, respectively.

*Class Content Interaction* The interactions with the Class content are shown in Fig. 2 on the right from bottom to top: The user’s *Virtual Character* can add and delete fields and operations on a Class side with her/his hands and use the *VR Text Input* component to change the textual content of these Class elements. All the elements on all Class sides exist within the *Class Content System* which captures changes and synchronizes them across the network via the *Event Synchronization Service*.

**Fig. 5:**
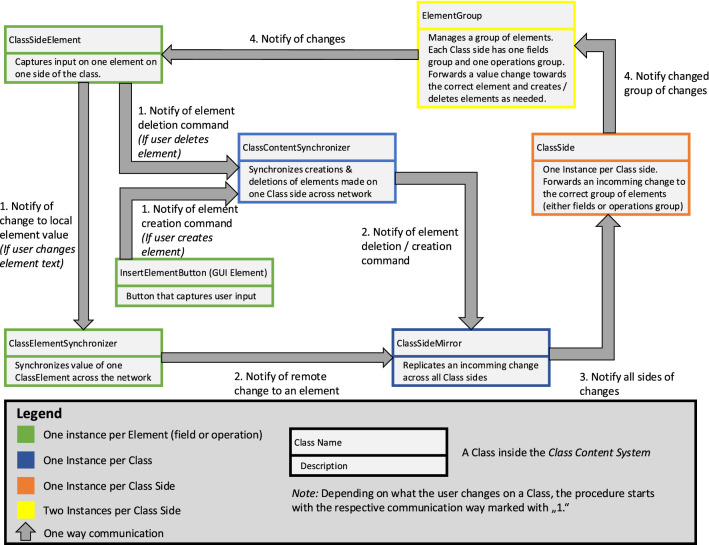
The architecture of the class content synchronization system

Since the actual content of a Class still consists of 2D elements, primarily text, on a Cube’s side, it is visually not very different from GUI elements. It is, therefore, logical to use the same general interaction concept that users are already familiar with from other GUIs for the Class content as well. Thus, laser pointer-based controls are used. This means that input fields like the Class name, for example, can be selected by pointing the right hand at it and pressing a dedicated “select” or “enter” button, analogously to what the left mouse button is on traditional input devices.

As mentioned before, the name is only one type of text in a UML Class that can be edited. The other major types are fields and operations. Some 2D tools, like Lucidchart [26] and Microsoft Visio [30], represent the two regions in a Class where fields and operations can be added as one input field each that spans multiple lines. Others let the user add fields and operations individually where each one is represented by a single line input field, possibly with additional control elements in that line.

This decision has some implications on how much collaboration might be possible during the creation of one Class. If two users want to simultaneously add different fields to a Class, for example, to split up the work, using a single input field for all fields could result in synchronization problems because both users are editing the same input field at the same time. This is especially relevant to our VR solution since it allows multiple users to stand in front of different sides of the Class and edit fields and operations on it that are then mirrored to the other sides. Therefore, we chose a single input line per field/operation approach, so each user can independently of each other edit such an element, even if they belong to the same Class.

These controls enable the user to create models collaboratively while always being able to see where the other collaborators are and what they are changing on the model.

*Class Content Synchronization* The *Class Content System* from Fig. 2 deals with the synchronization of the Classes’ contents and is largely independent of other parts of the solution. It is controlled by the GUI elements that exist on each Class side and only changes these elements and their values through the synchronization procedure. The architecture and workflow within this system are shown in Fig. 5. The figure is a customized version of a UML Class Diagram where the Classes have small descriptions instead of fields or operations and instead of relations, the communication paths between the Classes are displayed. The figure visualizes the modeling procedure that is executed inside the *3D Model* which is the target component in this case. Here, the *Virtual Character* serves as the bridge between the *VR Device* that tracks the user’s physical input and the GUI of the Class which provides an interface to the Class content of the *3D Model* to the *Virtual Character*.

Each Class inside the modeling environment has its own version of the *Class Content System* as shown in Fig. 5. Every Class has one *ClassContentSynchronizer* that internally uses the *Event Synchronization Service* to synchronize the Class’s content structure across the network. Each Class also has one *SideMirror* that mirrors the content structure across all sides of the Class. Each *ClassSide* is a container for that side’s two *ElementGroups* and the Class name. The Class name is directly managed internally by the *ClassSide* and therefore omitted in Fig. 5.

Each *ElementGroup* is either the group of fields that is displayed below the name of a Class in UML or the group of operations that is displayed below the fields group. Each *ClassSide* always has one *ElementGroup* for its fields and one for its operations. These *ElementGroups* each manage a list of *ClassSideElements* (i.e., either fields or operations, depending on the group) that exist in it.

A *ClassSideElement* is always one line in the UML Class where either a field or operation is displayed. This line always consists of an input field holding the text value of the line and a “delete” button that can be used to remove the respective element. Each *ClassSideElement* is responsible for capturing changes to the element’s input field and presses of the “delete” button. The *ClassElementSynchronizer* internally uses the *Event Synchronization* for synchronizing the changes to that input field’s text across the network. Every element (field or operation) on each *ClassSide* holds one instance of *ClassSideElement* and one *ClassElementSynchronizer*.

There are four types of changes that can happen to the content on one side of the Class that need to be synchronized across the other Class sides and across the network. These change types are: (1) A field or operation’s text value changing, (2) a specific field or operation being deleted (3) a new field or operation being created at a specific position, and (4) the name of the Class changing. Depending on which of these changes happened, the procedure of how the change is synchronized, as shown in Fig. 5, starts slightly differently.

Synchronization inside the respective Classes in Fig. 5 is always handled via the *Event Synchronization Service* (see Fig. 2). The actual workflow of how these events are used by the classes in Fig. 5 to synchronize changes across Class sides and across the network depends on what kind of change is happening.

Each change—independent of the type—always results from the user interacting with the GUI on one side of the Class. Changes to the input field of an element are detected by the *ClassSideElement* and forwarded to the *ClassElementSynchronizer* which synchronizes the change of the respective element with the other *ClassElementSynchronizer*s on the other clients through the *Event Synchronization Service*. On every client, the *ClassElementSynchronizer* receiving the event then notifies the *ClassSideMirror* of the Class to apply the change to the respective element across all Class sides.

On the other hand, when a user presses the delete button on an element, the element is not locally deleted but a deletion command for that element is forwarded by the *ClassSideElement* to the *ClassContentSynchronizer*. Similarly, when the user adds a Class element on a Class side, a creation command for an element at the specified position is forwarded to the *ClassContentSynchronizer*.

The *ClassContentSynchronizer* holds a model of which elements are on the Class in which order as two separate lists: one for fields and one for operations. When a deletion or creation command is sent to the *ClassContentSynchronizer* it edits this internal model and synchronizes the new version to its counterparts on the other clients.

This way, one content synchronization event always contains the complete current structure of elements on the Class, meaning that old events do not need to be saved. If each event would only contain one specific change to the structure (like addition or deletion) all previous events would be needed to arrive at the correct structure. This is important since clients that join a room later need to be able to recreate every Class’s structure easily so their Classes are synchronous with the ones of the clients that were already in the room. With this system, the new clients only need to be passed the most recent content synchronization event by the *Event Synchronization Service* to bring them up to date.

These change events are triggered by one client and sent to all clients, including itself, so all of them start the procedure of changing their model elements and the visual elements at approximately the same time. When such an event is received each of the *ClassContentSynchronizer*s on the different clients calculates what changes need to be made to transform their own (now deprecated) model into the newly received one. It then executes those changes on the model and forwards the appropriate commands to the *ClassSideMirror*, so it can bring the visual representation on the *ClassSide*s in line with the now updated elements model.

**Fig. 6:**
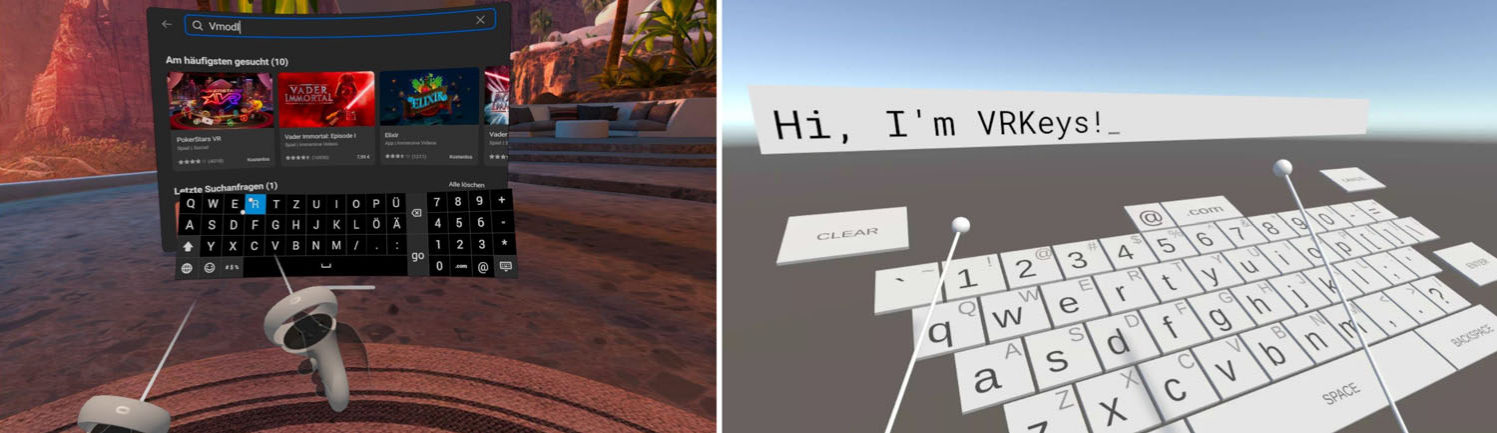
Examples of VR keyboards: The Meta Quest 2’s Laser pointer-style keyboard (left) and the drum-style keyboard *VRKeys* (right) [46]

It is important to understand that the *ClassContentSynchronizer* only synchronizes the structure of the elements without their textual content. The contents are synchronized via the *ClassElementSynchronizer*s as discussed before and then forwarded to the *ClassSideMirror*. This separation is done to reduce the amount of data that needs to be sent across the network. Since the Class content events hold the entire content, instead of just the changes to the content, if the textual content of each element would be included in it, then for each text change (i.e., each time any letter changes in any of the Class’s elements) the entire content of the Class would need to be transmitted. Therefore, structural and textual changes are separated from each other because the textual ones will happen a lot more often than structural changes.

Once the information about a change has been synchronized across the network, the *ClassSideMirror* is always notified of them, so it can change the visual representation on the *ClassSide*s accordingly. The *ClassSideMirror* holds a list of all six *ClassSide*s and simply replicates the change instructions across all of them. Each *ClassSide* receives these instructions and, based on whether a field or operation should be changed, forwards them to the respective *ElementGroup*. This group then creates or deletes an element according to the instructions or—in the case of a textual change in a specific element—forwards the instruction to the correct *ClassSideElement*. The *ClassSideElement* then finally updates the text inside the element’s input field but without triggering a new synchronization call to the *ClassElementSynchronizer* to prevent an infinite loop.

Changes in Class names are handled largely the same way as changes to Class elements since all of them are primarily input fields with text values that need to be synchronized across all sides and across all clients. The only real differences between them are that Class names—unlike elements—cannot be deleted or created and exist exactly once per side and thus do not need to be grouped into *ElementGroup*s. This means that a *ClassSide* can directly change the value of its Class name input field when it receives a change notification from the *ClassSideMirror*. It does not have to go through a *ClassElementGroup* first. Otherwise, the synchronization works analogously.

This structure ensures a strict separation of concerns between the individual parts inside the *Class Content System* that are displayed in Fig. 5. Simultaneously, it makes sure that changes happen on all clients as synchronously as possible to avoid inconsistency within a single Class across multiple clients when multiple users edit that Class at the same time.

### VR text input

In VR, text input is more difficult compared to traditional computers due to the lack of a physical keyboard that provides haptic feedback on whether a key was hit or not. Since the user cannot see and use a real keyboard in VR, virtual keyboards are often used instead. As shown in Fig. 6, there are two basic types of keyboards regularly found in VR applications: Laser pointer and drum-style keyboards.

Laser pointer-style keyboards are usually displayed as a vertical plane in front of the user with each hand representing one laser pointer that can be used to press a key with a dedicated button. Since the buttons have to be rather large to be easily hittable, this input method requires a lot of space. An alternative to laser pointer keyboards are drum-style keyboards which we have chosen in our solution (see Fig. 8b). These are shown in a horizontal, slightly tilted form in front of the user like many real keyboards are as well. One virtual mallet is attached to each of the user’s hands that can be used to hit a key similar to how a person would hit a drum in the real world. Because of the obvious drum analogy, it can be assumed that this control scheme is also easy to understand for most users and could be more natural to use than laser pointers. Additionally, the profile of the keyboard is more flatter than laser pointer-based ones, so a user can easily edit text that is displayed upright in front of her/him while the keyboard occludes less space in the front and to the sides of the user. For these reasons, we use a drum-style keyboard as the primary method for *VR Text Input* inside our solution.

**Fig. 7:**
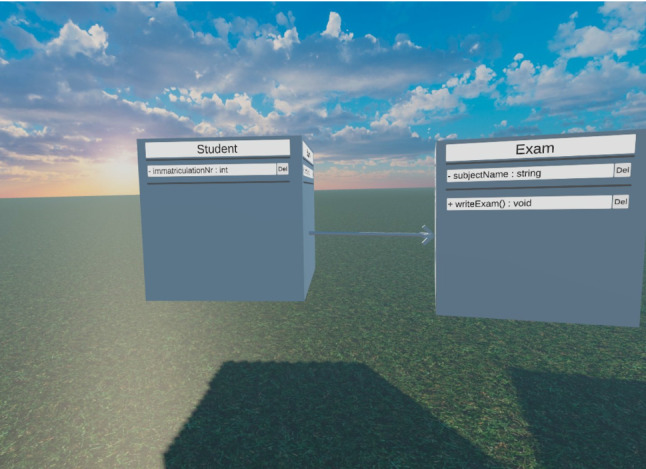
An example UML Class Diagram with our custom 3D representation

However, VR keyboards are sometimes not the only method to enter text. The Meta Quest’s YouTube app, for example, allows the user to enter search terms by speech recognition, making it substantially easier to use than virtual keyboards, provided the recognition algorithm works correctly. While such speech recognition can work very well for standard sentences, text in UML Class Diagrams is notably different in that it uses a lot of special characters like colons and braces and has its own grammar rules when it comes to things like word separation and capitalization. In a UML Class Diagram words inside a field name, for example, are not separated by white spaces but by a capital letter for each new word while the first word is usually started in lowercase. Since these special cases are not easily reproducible with modern speech recognition, we chose not to include such an option in the solution and focus on the keyboard method instead.

## Implementation

In this section, the implementation of our collaborative modeling environment in VR is described based on the conceptual overview (see Fig. 1) that was presented in the previous section. The source code of our collaborative modeling environment VmodlR is published via GitHub^4^ and open for public access.

### VR devices

The *VR Device* is a central part of the implementation and conceptual solution, as shown in Fig. 1 because it is the gateway of every *User* into the *Virtual Environment* where all interactions—with the model and with other *User*s—take place. The *VR Device* also largely dictates the technological possibilities of the implementation and therefore it is important to decide about this component.

We wanted to focus on one headset because this typically decreases development time compared to supporting multiple platforms. Since one goal of the conceptual solution was to provide interactions as natural as possible to users, we needed a target device that supports the features like hand presence support. The most common way for *VR Device*s to provide this specific feature are two single-handed controllers provided with the devices. Additionally, we needed the headsets to function on their own with tracking in six degrees of freedom and not require elaborate setup, like outside tracking sensors, so users can quickly pick up and use the system without being bound to a specific location. Therefore, we needed a stand-alone headset with inside-out tracking and two included single-handed controllers. The last factor in our decision was the device cost. We wanted to make it as realistic as possible that companies or educational institutions would buy multiple headsets so software engineers could collaborate in VR, meaning that these headsets should have a reasonable price.

For all these reasons, we chose the Meta Quest 2 as a target implementation platform. The Quest 2 is a cable-free, mobile VR HMD that includes all the above features while enabling the user to move around on a room scale with full Six degrees of freedom without the need to set up additional sensors before using it. It also has a low price compared to other VR headsets.

### Virtual environment

The *Virtual Environment* is the basis of this solution since every experience a *User* has inside the application takes place inside the *Virtual Environment*.

For the implementation of the VR environment, we have used the Unity 3D Game Engine developed by Unity Technologies [47] as it includes a fully-featured Graphics, Sound, and Physics Engine and has easy-to-use APIs for many different aspects like controller input. Furthermore, Unity also provides support for the Meta Quest 2 among other VR headsets through an Oculus VR SDK.

Concerning the design of the *Virtual Environment*, we had two important requirements that were introduced before: (1) realism, for maximized naturalness and immersion, and (2) practicality for 3D modeling. To fulfill these requirements, we relied on free online sources like the Unity Assets Store where many rather high-fidelity assets can be downloaded free of charge.

The grass floor is a repeating texture without actual 3D grass to spare the limited resources of the Meta Quest’s mobile processor. The sky is a *skybox* that is usually used to simulate a sky in Unity that was downloaded from the Unity Asset Store. This skybox depicts a slightly clouded blue sky with a setting sun. To make the sun displayed in the skybox actually cast shadows onto the world, we positioned a directional light accordingly which is a virtual light source included in Unity that is regularly used to simulate sunlight. Figure 7 shows the resulting *Virtual Environment* with some objects that cast shadows according to the sun’s position.

### Network synchronization

To enable remote collaboration, a networking system is needed that synchronizes aspects like model element- and user avatar positions, user hand gestures, etc. For the implementation of the networking system, we have used the Photon Unity Networking 2 (PUN2) plug-in for Unity. This is a third-party system developed by Exit Games specifically for use in Unity multiplayer projects and offers easy-to-use high-level components. PUN2 realizes the network architecture discussed in the previous section by providing its own *Cloud* servers that automatically work with PUN2 without the need for any custom server-side development.

### Virtual character

**Fig. 8:**
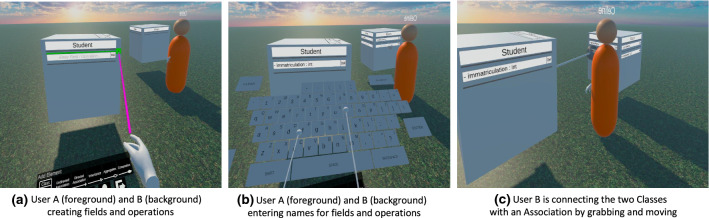
Screenshots of the collaborative VR modeling environment

The Oculus Software Development Kit (SDK), used to realize the interaction between the *VR Device* and the *Virtual Environment*, comes with a variety of assets that can be used to quickly implement common functionalities inside a VR application. Most aspects needed to realize the *Virtual Character* are covered by those assets provided by Oculus. Therefore, to save development time on those basics, we used Oculus’ *OVRAvatar* and *OVRCameraRig* assets and adjusted them slightly for our application. The OVRCameraRig deals with tracking the position and rotation of the head and controllers and rendering the world accordingly into the headset. It does not include animated hands by default but there are separate assets for that which can simply be placed under the empty GameObjects that track the controller positions to enable users to see their hands. The gestures of the hands are tracked locally by default through an animator so we only had to add an animator synchronizer provided by PUN2 to synchronize the hand’s animation states across the network [14]. Since all *Virtual Characters* are represented equally in this prototype, we chose to display every user’s name above their character. An example of how a remote user’s *Virtual Character* looks inside the VR app is shown in Fig. 8.

**Fig. 9:**
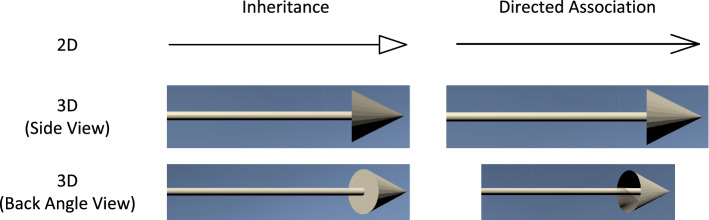
Comparison of inheritance and directed association when using 2D bodies of rotation to create the 3D designs

**Fig. 10:**
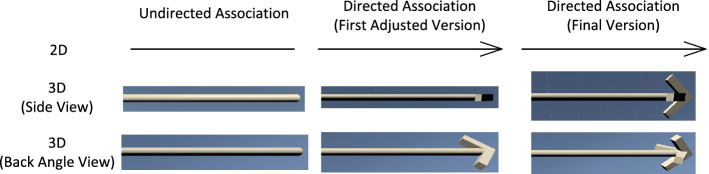
Comparison between the undirected association and the directed association with the first adjusted and final 3D representation versions

**Fig. 11:**
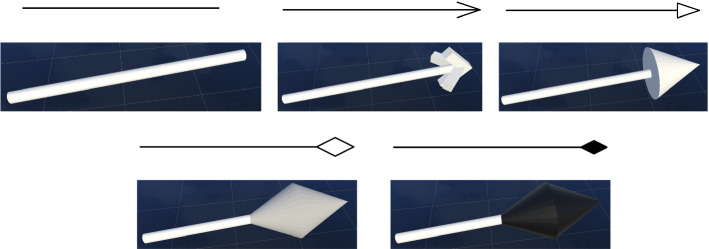
Pairwise 2D (top) and 3D (bottom) designs of the supported arrow shapes from UML Class Diagrams

### 3D models

In the following, we will describe the implementation of the *3D UML Visualization* and *Model Editing*.

#### 3D UML visualization

As outlined in the conception section before, the main goal of the *3D UML Visualization* is to provide a 3D adaptation of the standard 2D UML shapes that will seem natural to users in the sense that a user, only familiar with the 2D shapes, can easily recognize their 3D versions.

One method to find natural equivalents of 2D shapes in 3D is to use the 2D shapes as bodies of rotation to generate a 3D shape. This means the 2D shape is turned around an axis in 3D space and the trail it leaves while turning is the 3D shape. In the case of arrowheads, the connection’s line can be used as the axis to turn around. However, this results in some of those shapes looking rather similar to each other. This is especially critical since, in 3D, they can be viewed from different angles, and regardless of the viewing angle, a user should always be able to easily identify each element.

For example, using the body of rotation method, an Inheritance would look rather similar to a directed Association from the side. Only when viewed from behind, it is perceivable that the Association’s arrow is hollow while the Inheritance arrow is solid. This is shown in Fig. 9.

To circumvents such issues, we took the rotational bodies only as starting points to make sure the shapes are recognizable enough for software engineers used to the 2D visual language. We then adjusted these initial designs to make them better distinguishable. In this case, we adjusted the representation of the directed Association to use an arrow shape consisting of two cuboids. However, this version has another problem: When it is viewed from the side that the cuboid points in, it can be hard to distinguish from undirected Associations. This is shown in Fig. 10.

Therefore, we added another arrow shape to the tip of the directed association that is positioned at a $90^{\circ }$ angle to the first arrow shape. This way, the directed association can be easily identified independent of the viewing angle. The resulting set of connections with their different arrow heads that were used in our collaborative modeling environment can be seen in Fig. 11.

#### Model editing

In the following, editing the *3D UML Visualization*, i.e., the model, and synchronizing its state across the network will be described in more detail.

**Fig. 12:**
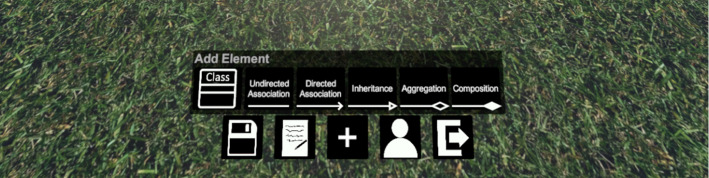
The primary menu while modeling, with an expanded *Add Element* panel

***Creating and Deleting Elements:*** Every user’s *Virtual Character* has a *Creation Menu* following it around through which the user can create new model elements by pressing GUI buttons with laser pointer style controls. Figure 12 shows how this *Creation Menu* was realized in the prototype. Besides the *Creation Menu*, this menu implementation also includes some additional features that can be accessed via dedicated buttons. This was done in a way that the user only has one menu through which all functionality that requires a menu (e.g., leaving the room) is accessible.

To reduce the overall space the menu takes up, we decided to only display a small number of icons through which additional panels can be opened and closed. For example, the “+” button in the center opens and closes the “Add Element”-panel, i.e., the *Creation Menu*, through which new UML model elements can be created. Figure 12 shows the menu with an expanded “Add Element”-panel. Other icons open panels for saving and loading of models, a task description for the usability study that will be discussed in the next section, and a list of users in the current session, while the final button lets the user exit the current modeling session.

The *Creation Menu* includes dedicated buttons for all supported model elements which are labeled textually and with the respective 2D UML element so users can easily remember which button creates which model element. These GUIs, like all GUIs in the application, were implemented using the Unity Engine’s built-in GUI system which provides basic elements like panels, input fields, and buttons that can be arranged to form menus in a visual editor and can be equipped with functionality through custom scripts. The Oculus SDK provides support for this GUI system through pre-made assets that implement laser pointer style controls for the GUI without a need to adjust the individual GUI elements.

Whenever one of the buttons for creating a model element is pressed, a prefab of that element is created through a creation method provided by PUN2. This creation method is ensured to be called on the master client because this is the client responsible for creating and deleting room objects, i.e., objects that should persist even when the client that spawned them leave the room.

For this, a Remote Procedure Call (RPC) is sent from the client that pressed the respective “create” button to the master client which then handles the instantiation of the model element’s prefab through the creation method provided by PUN2. Since the given prefabs include Photon View Components, PUN2 then handles all synchronization of these objects automatically, including the creation of these objects for later joining clients.

Deletion across the network works rather similarly. A user can toggle a red deletion laser pointer by which the user enters or exits the deletion mode. The object, the laser pointer points at, gets marked red and the user can press the “select” button—usually used to press a GUI button—to delete it.

Internally, each model element holds a script that marks it as a *Deletable Element*. The laser pointer then checks if it is pointing at an element with this script when the user presses the “select” button. If such an element is hit, the object is deleted over the network through an analogous procedure to an object’s creation. Therefore, once the delete-method was forwarded from the master client to PUN2, Photon again handles all networking tasks internally, like it did when creating a new model element.

This system offers a simple setup for efficiently creating and deleting the networked model elements through the PUN2 networking infrastructure and should be easy to use for the application’s users, aligning with this paper’s goal of providing remote collaboration support in a 3D modeling environment in a natural and intuitive way.

***Moving Model Elements:*** As described earlier, the user can move model elements by grabbing objects with the *Hands* of the *Virtual Character*. With the Oculus SDK, there is a grabbing system included which provides exactly the functionality we need without having to write much additional code. The system works by having an *OVRGrabber* script attached to each *Hand* and an *OVRGrabbable* script added to all objects that it should be able to grab, hold, and move, like the Class cuboids. This way, the Classes can be grabbed anywhere by the Cuboid that visualizes it while the Connections have dedicated *GrabVolume*s at each end that the user can grab and move. These GrabVolumes were implemented as spheres in this prototype.

**Fig. 13:**
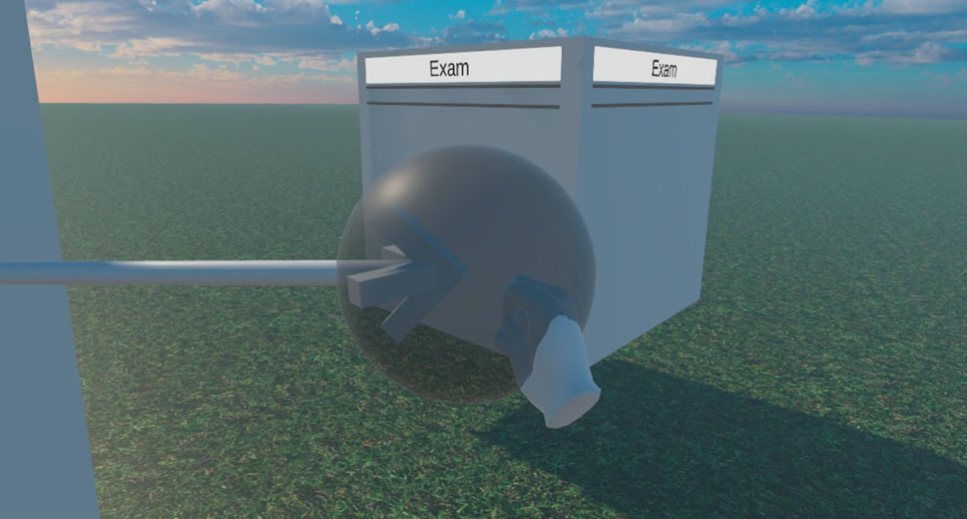
A user grabbing and moving one end of a connection

To make it easy to grab these spheres, they are represented rather large in the VR application. Large GrabVolumes bring the problem that they can occlude important parts of the model, crucially the arrowheads that are necessary to differentiate the Connection types. Therefore, we made the GrabVolumes partly transparent and let them only display when a user moves one of their hands toward the start or end of such a Connection. This makes it easy for the user to see if the Connection can be grabbed at the point where the user’s hand currently is, while simultaneously not occluding important parts of the model when the user does not want to move the Connection. Figure 13 shows a user grabbing a GrabVolume in the VR application.

***Editing Class Content:*** In the previous section, we have already discussed how the editing and synchronization of content inside a modeled Class were designed through a setup of classes communicating in a predefined way (see Fig. 5). These classes were implemented in the prototype through scripts that are placed on the Class GameObject and its child objects, e.g., the *Class Side*s.

The synchronization inside the *Class Content System*’s classes is always handled via the Networked Events with a dedicated event type for each script class. In addition to the event type, the event also always has to contain the ID of the Class or *ClassSideElement* that the event is about, so changes are only applied to the correct element inside the correct Class.

In the case of the Classes, Photon ViewIDs are used for this, because they already are ensured to be unique inside a room by PUN2. Elements have specialized IDs. Each element ID is not only unique within a Class but also unique across all Classes in a room. This makes it easier to ensure that the IDs of elements are unique because only one centralized networked variable (called room property in PUN2) can be used to represent the next higher ID that is still free. When a new element is added, it tries to get a new ID by changing this room property and if no two elements try this at the same time, the element receives the previous value of the room property as its new ID. In this procedure, two elements may clash because they try to set the ID property at the same time to the same higher value. This is serialized by one element being given the new ID and the other element retrying the process with the next higher ID.

**Fig. 14:**
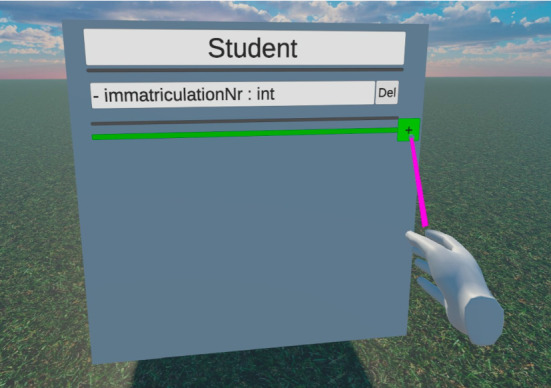
Inserting a field or operation through the Button-Line-Control

This provides an efficient implementation of the *Class Content System* through C# scripts in Unity and PUN2’s Network Events. The actual GUI elements on the Class Sides, e.g., the input fields for Class names, are realized just like the GUIs that are used for the *Virtual Character*’s *Creation Menu*. The user can delete elements from a Class through a dedicated “Del” button for each element as shown in Fig. 14 on the right of the Class.

Adding of elements was realized by displaying a green insertion line when the user aims above or below an element with their hand. A larger green “+” button is also displayed on the line’s right end. This is shown in Fig. 14. The user can press either the line or the button to insert a new element, and the elements disappear when the user is not aiming at one of them.

This combination of an “insert” line and button strikes a balance between the granularity of being able to insert a new element anywhere on a Class and the difficulty to hit a thin line without the larger button by pointing at it.

### VR text input

The *VR Text Input* component from Fig. 1 was realized as a drum-style keyboard in this solution. This choice was already motivated in the previous section. In the following, we will describe how this keyboard was implemented in the VR application.

**Fig. 15:**
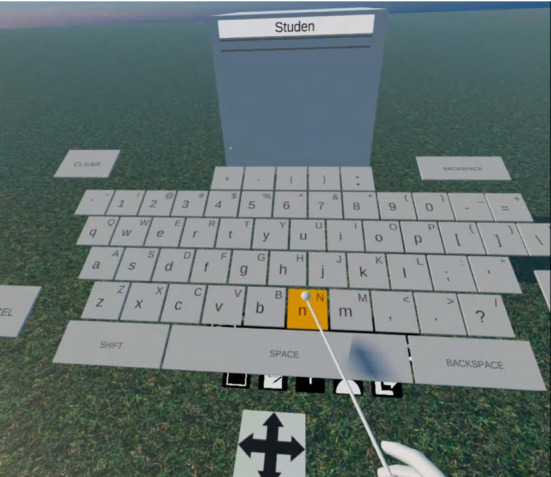
A user operating the drum-style keyboard in VR

Since there are several prebuilt solutions for these types of VR keyboards available on the Unity Asset Store that fulfill their task adequately, we chose to implement the keyboard based on such an existing solution. Thus, we chose the *VRKeys* package from the Unity Asset Store [46] since it is free, provides a customizable keyboard layout, and offered user feedback on whether a key was hit or not: When a user hits a key with a mallet, the key moves down (like a key on a normal keyboard would when pressed), it makes a clicking sound, the controller that was used to press the key slightly vibrates and the key turns yellow for a short period. This means that there is audio, visual, and haptic feedback provided with a keystroke out of the box which can be assumed to help the user notice which key was pressed and alerts the user if an accidental keystroke happened. Figure 15 shows this drum-style keyboard as implemented in the prototype.

One important aspect that had to be adjusted in the keyboard to make it work in our VR application was setting up the connection between the input fields, like Class names, and the keystrokes.

By default, the keyboard directly edits an input field attached to the top of it. Since we needed to make it work with arbitrary input fields on Classes and in the GUI we had to find a way to make it appear whenever the user selects an input field and make the keyboard edit that input field. The currently selected GameObject is always tracked by Unity in an *EventSystem* object which is primarily needed to control GUIs since interactions with these are handled via local events in Unity. Therefore, by hooking into the *EventSystem* and checking if a newly selected GameObject is an input field whenever the selection changes, the keyboard can be immediately opened and closed whenever it is requested through a click on an input field. This enables a reference to that input field to be passed to the keyboard whenever it is enabled, without the need to set up every input field with a reference to the keyboard. After the keyboard is displayed and has been given a reference to the currently selected input field, it changes its content according to the keystrokes.

The management part of interfacing with the *EventSystem*, passing the correct reference to the keyboard and activating or deactivating the keyboard accordingly is handled by a dedicated *KeyboardManager* class while the input handling and changing of the input field once the reference is setup is handled in the *Keyboard* class to ensure proper separation of concerns.

With this system, any text input where GUIs are involved can be handled through the GUI’s *EventSystem* and the keyboard classes without any further setup required for dynamically created GUIs, like the ones found on Classes.

## Evaluation

To evaluate the efficiency, effectiveness, and user satisfaction of *VmodlR* and to compare it with conventional collaborative modeling approaches, we have conducted a user study which will be presented in the following.

### Setup and participants

The user study was organized in such a way that two people in different rooms had to collaboratively create a UML Class Diagram. We have chosen a within-subjects design [3] for our user study where the participants were asked to use both modeling approaches, a conventional modeling tool, and the developed VR modeling tool. Two different small UML Class Diagram modeling tasks (consisting of five classes and based on a textual description) were provided to the participants, while the sequence and type of task which was carried out with the help of a modeling tool were evenly distributed to avoid potential bias in the collected data. As a reference application for comparing with our own VR environment, we have decided to use an existing commercially available tool for collaborative UML modeling. The tool we used is the web application Lucidchart [26]. This was chosen because it offers a mode of collaboration many users are already familiar with from services like Google Docs, it offers a free version that could be used for this study, and supported UML Class Diagrams. The evaluated applications focus on remote collaboration, therefore, we simulated a remote setting by placing each participant in a different room during their collaborative tasks where they could only communicate through computing devices (conventional and VR device).

For the VR application, each participant was positioned in a free space of approximately the same size and equipped with an Meta Quest 2 VR Headset including its two VR Controllers. The participants were able to communicate over the application’s voice chat feature using the Meta Quest’s built-in microphone and speakers. The respective task was displayed inside the VR app on a panel that could be opened and closed through an icon on the user’s menu. The panel was positioned slightly to the left of the user so they could leave the panel open and edit the model in front of them at the same time. While solving the web task, each participant was sitting at a desk in the same room that the VR free space was in, equipped with a laptop and a wired mouse. The participants could use the laptop’s keyboard, its trackpad, and the mouse as they saw fit. Since the web application does not include voice chat, the participants communicated via the voice chat application Skype that was running in the background on their laptops. The app used the built-in speakers and microphones of the laptops. The tasks were supplied to users on a sheet of paper so, like in the VR app, it would not occlude their modeling environment unnecessarily.

**Fig. 16:**
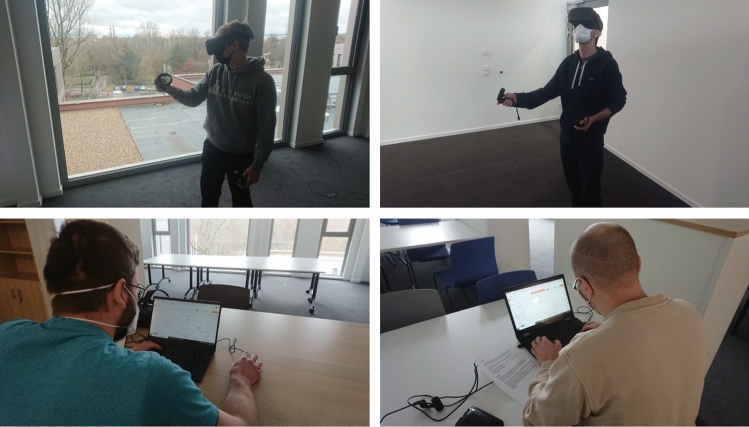
Users participating in the study. Two collaborators using the VR app (top) and the web app (bottom)

Figure 16 shows the basic setup of our user study where two participants are collaborating in VR (top) and through the web app (bottom).

Since this study involved participants having to create two small Class Diagrams, these participants had to bring a basic knowledge about what a Class Diagram is and which purpose it serves for software modeling. Therefore, we had to rely on participants who either had lectures or school classes on this topic, for example, in computer science lectures or subjects and/or who knew Class Diagrams from a different source, like working as a software developer who uses them. Therefore, we primarily tried to acquire participants with educational backgrounds, like university students and recent graduates. Our main source was a lecture on Model-Based Software Engineering designed for undergraduate students of computer science in their third year of studies. In this lecture, we presented the study and asked students to participate in it. We also reached out to students we were still in contact with who took part in this lecture during the prior year to widen our pool of possible participants while still ensuring comparable credentials among them. In total, 24 participants took part in the user study, meaning that there were 12 groups of two people each.

### Procedure

The user study was conducted during one week in February 2021. The experimental setting was kept as equal as possible for all pairs of participants. First, the participants were greeted and introduced to the user study. Then, the basic procedure of how they will take part in the study and what they will do during their participation were explained. Afterward, depending on whether the VR or web app was used first, the collaboration environment was set up (e.g., splitting the participants, starting Skype, etc.), and they received a short introduction to the respective program. In the case of the web application, this was done through the study supervisor explaining the main functionality from a pre-written script to ensure all participants were given the same information. In the VR app, we have additionally implemented a tutorial that served as an on-boarding tutorial at the beginning of the user study. After the tutorials, the participants were provided with the respective task and instructed to solve the task collaboratively in the sense that they should only create one Class Diagram together in each application. They were then asked to indicate to the study executor once they think they are done with their task. When the participants finished the first task, the procedure was repeated for the second task. After both tasks were finished, the collaborative applications were closed so participants would not be able to talk to each other anymore. They were then given the questionnaire hosted via Google Forms [17] and were asked to fill it out using the laptops that were used for the web application as well. During this process, the users stayed in the different rooms they were in while working on the tasks to ensure that they would not influence each other’s answers.

### Usability measurements

**Fig. 17:**
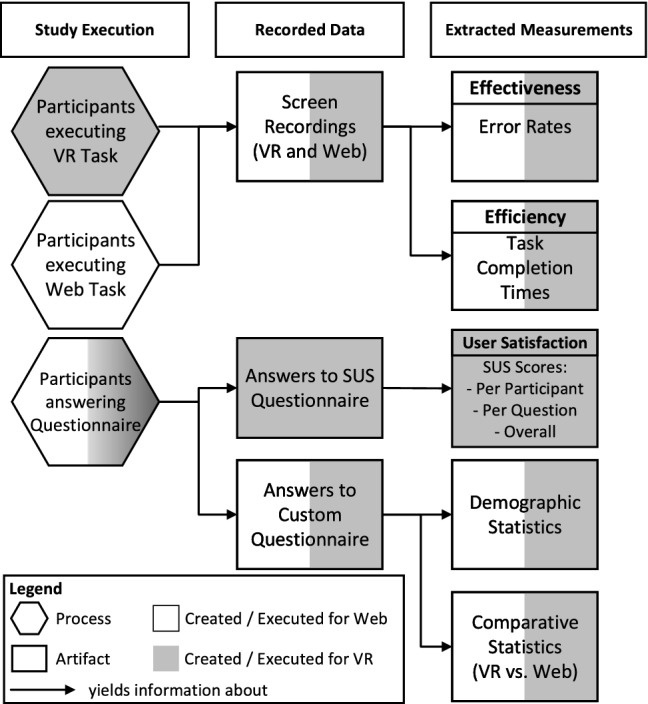
An overview of what was measured within the user study

To extract meaningful results from the study, we had to choose certain measurements that we would take during the execution. Figure 17 shows an overview of the data that was collected during the user study and which measurements were derived from that.

**Fig. 18:**
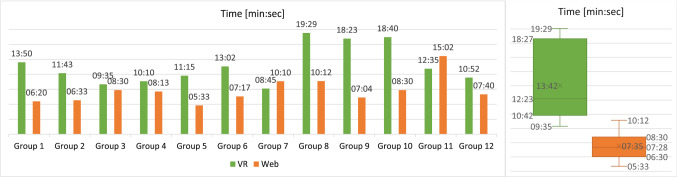
The times each group took to execute the task on the web and the VR app, respectively

Efficiency was measured by recording the participants’ execution of the task and tracking the time from the point where they started reading the task to the time when they told the study executor that they finished it. From these same recordings, the effectiveness was measured by counting the number of operating errors that the participants made during the execution of the task. From this, an error rate was calculated by dividing the number of errors by the time in minutes that was measured as the efficiency. This error rate was our final score for the effectiveness in errors per minute. The recordings used for tracking efficiency and effectiveness were screen-captures including the voice chat audio. In each group, only one participant was recorded to reduce the amount of video data that had to be manually evaluated. Another reason for that was the network infrastructure that did not allow us to capture and record two video feeds from the VR headsets in parallel. The user satisfaction was evaluated through a questionnaire that participants were asked to fill out after they finished both tasks. We chose to use the System Usability Scale (SUS) questionnaire [9] since it is a well-proven and reliable questionnaire that provides comparability with SUS evaluations of other applications and that can be quickly filled out due to its low number of questions [7]. While efficiency and effectiveness were measured for both applications, the SUS questionnaire was only asked in the context of the VR application. This is because the SUS questionnaire’s main purpose in this work is to provide a proof of concept that the VR solution has rather good usability. Furthermore, the participants were asked to answer a custom-developed questionnaire that included mainly 5-point Likert scale questions (like the SUS questions) and some free text questions asking for more specific impressions with regard to collaboration and interaction.

### Results

In the following, we present the main results of the user study.

#### Demographic statistics

Twenty-four people in 12 collaborating groups participated in the study. All participants were between 22 and 30 with a median of 25 and a mean of 25.5. Twenty-one of the 24 participants were male and the others female. Most of the participants were software engineering practitioners or had a background in computer science. As a consequence, many participants reported advanced experience with UML and UML modeling tools. The average rating scores for prior experience with UML and UML modeling tools were 3.25 and approximately 3, respectively. With regard to prior experience with VR, our results show that half of the participants have never used a VR device before this study while 8 used them at least reasonably often.

#### Efficiency

The times participants needed to complete their tasks are shown in Fig. 18.

All but two groups (Group 7 and 11) needed more time in VR than in the web application to complete their task. From the recordings, it could be observed that almost all groups split up the work on the Class Diagram by each modeling one part of the diagram and putting both parts together in the end. During this process, they occasionally discussed how to model certain aspects if they were unsure and checked the part of the model their collaborator created in the end. The two groups that took longer in the web application than in VR had a slightly different approach that could explain this anomaly: In VR, they split up their task as outlined above. In the web application, however, they mainly followed a pair-programming style approach: For each part of the task they discussed how to model it, and then only one of them modeled the aspect accordingly. The first approach obviously saves time in comparison with the latter one which could explain why they were able to complete the task faster in VR than in the web application when everyone else needed longer in VR. This makes the times for these two groups not comparable since they did not use a similar organizational method in both conditions. Therefore, we excluded them from time-related analyses like the box plots shown on the right of Fig. 18.

**Fig. 19:**
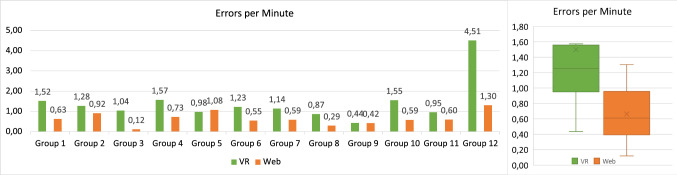
The error rates of the recorded participant in each group

Besides this anomaly, it can be said that the spread with respect to time was way smaller for the web application than for VR, i.e., they vary less as shown on the right of Fig. 18. In VR, times range from almost 20 minutes to as low as 9:30 min, while in the web condition, the times vary from approximately 5:30 to 10 minutes. The difference of each group between web and VR had a mean of 4:46min and a median of 5:26. This time-data may be normally distributed around its mean but does not have to be. To perform a significance test, we used the Wilcoxon Signed-Rank test [1] since it does not assume a normal distribution, and based on our test we cannot confirm that both samples are normally distributed. This test reports a $Z = -2,589$ with $p < .05$, meaning that VR had statistically significantly longer task times than web.

#### Effectiveness

In order to consistently track errors across all recordings, we had to define what “error” means in the context of this usability evaluation. Our definition of a *Usability Problem* is based on [29] where the authors define a *Usability Problem* as “a set of negative phenomena, such as user’s inability to reach his/her goal, inefficient interaction and/or user’s dissatisfaction, caused by a combination of user interface design factors and factors of usage context” [29]. Based on this definition, we extracted 3 types of errors that we analyzed in the recordings:

1. Missed Interaction Point: *The user tried to interact with an element of the application but did not hit the said element (for example a button) or used the wrong control for interaction. Thus, the user needs to repeat the interaction.*
2. Accidental Interaction: *The user did not intend to interact at all or not with this specific element but accidentally interacted with it anyway. Thus, the user needs to revert the interaction.*
3. False Interaction: *The user tried to do an interaction that is not possible at all or not possible at that specific element. Thus, the user experiences a loss of time and needs to find out the correct interaction to achieve the desired effect.*

During the evaluation of the recordings, we tracked each error by its type and a time stamp. Finally, the effectiveness was measured in errors per minute and is shown in Fig. 19. Most errors belonged to the *Missed Interaction Point* or *Accidental Interaction* types with only a few *False Interactions*.

In VR, we observed a mean error rate of approximately 1.5 (error per minute) with a median of 1.25 (error per minute), while these values lay at 0.66 and 0.61 (error per minute), respectively, in the web application. The Wilcoxon Signed-Rank test resulted in a test statistic of $Z = -2.599$ with a significance $< .05$ which shows that VR had a statistically significantly higher error rate than the web-based application.

#### User satisfaction

The basic user satisfaction was measured in the VR application using the 10 items SUS questionnaire for each participant. In total, our collaborative VR modeling environment reached an average SUS score of 78 out of 100 which indicates according to [43] good usability. As mentioned earlier, we did not ask the participants to fill out the SUS questionnaire for the web application as our focus was more on the acceptance of our own VR solution than on the acceptance of a mature and commercial modeling tool like Lucidchart.

#### Additional questionnaire results

Besides the presented usability evaluation metrics, we had further additional questions covering aspects like interactivity and co-presence, usage fun and motivation, intention to use the tools in the future, general application preference as well as open-ended questions to receive general feedback and remarks. In the following, the main results of these additional questions will be described.

**Fig. 20:**
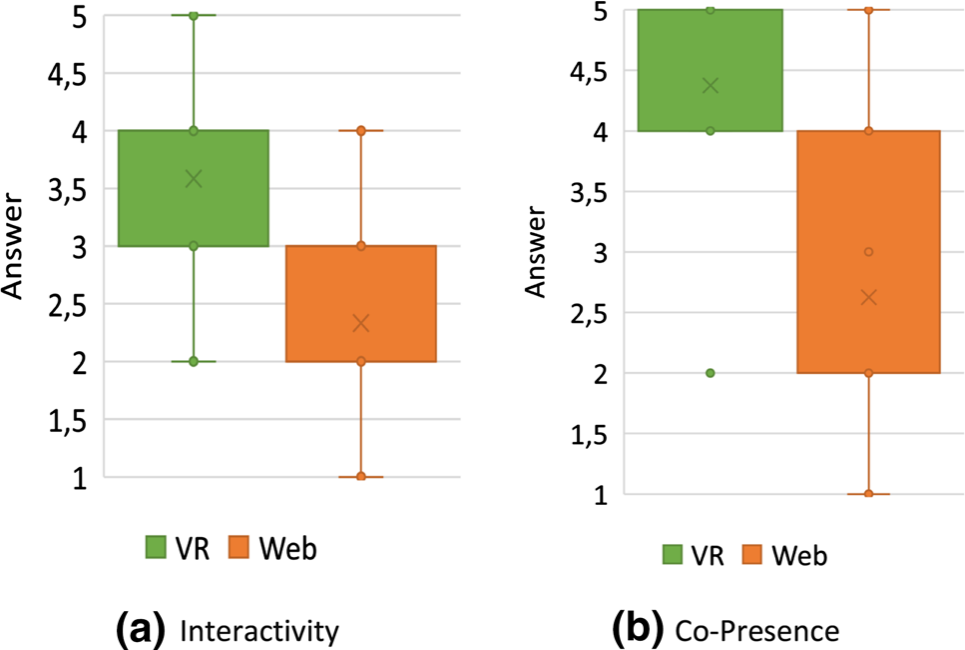
Results of additional questions concerning interactivity and co-presence

*Interactivity and Co-Presence* To assess the perceived naturalness of both applications, the web and our VR solution, the similarity of interactions inside the applications to face-to-face interactions and the feeling of co-presence were analyzed. The results are depicted in Fig. 20.

Concerning the similarity of interactions to face-to-face interactions (see Fig. 20a), the average score of the web application is 2.33 and noticeably lower than the VR’s 3.58. Additionally, in VR 14 out of 24 people selected the highest (5/5 on the Likert scale) answers whereas, in the web, nobody chose the highest and only two people chose the second highest answer (4/5 on the Likert scale). A Wilcoxon Signed-Rank test revealed that this observed difference is indeed statistically significant with $Z = -3.208$ and a significance of $< 0.05$. Overall, this means that users found interactions with their teammates to be significantly more similar to face-to-face interactions in the VR modeling environment compared to the web application.

A further important aspect for natural interaction and collaboration is co-presence which denotes the feeling of being in the same place despite the remote setting. Figure 20 (b) shows how the participants assessed their feeling of co-presence in both applications. It shows that in the web-based application, this feeling is very different from user to user, with larger bulks at both ends of the scale. The median, however, with a value of 2.6 points more toward the lower end of the scale. In the VR app, this is very different. While two people rated the co-presence rather low (2 on the 1-5 scale), every other participant at least somewhat agreed with the sentiment that there was a feeling of co-presence with 9 participants selecting 4/5 and 13 choosing 5/5. The Wilcoxon signed-rank test confirmed that this difference is statistically significant with a $Z = -3.587$ and a significance of $< 0.05$. This implies that the VR application can more successfully make users feel like they are collaborating in the same room compared to the web app.

*Usage fun and motivation* Previous research has shown that users find VR solutions to be more fun and motivating to use compared to traditional solutions based on desktop computers [36]. To assess if that translates to the collaborative modeling of UML Class Diagrams, we included two questions where users had to indicate if they found the VR or the web application more fun and more motivating to use, respectively.

**Fig. 21:**
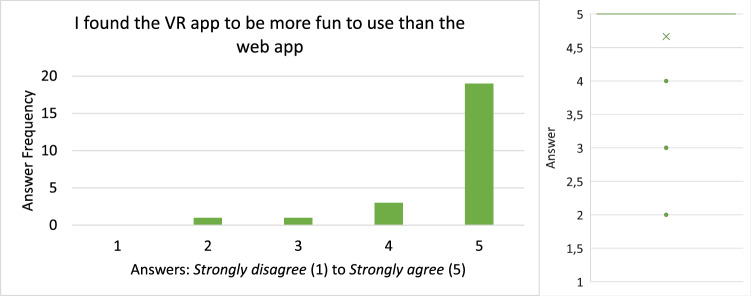
Participants’ fun using the applications

**Fig. 22:**
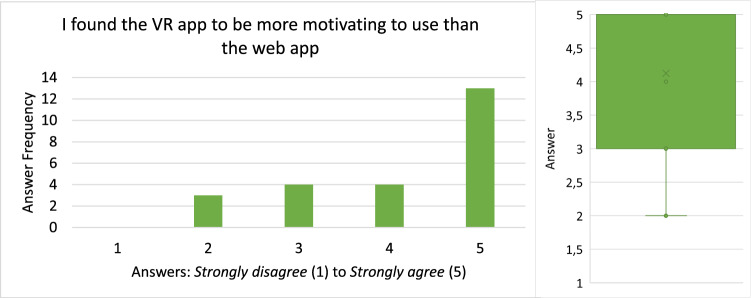
Participants’ motivation using the applications

The answers to these questions are visualized in Figs. 21 and 22. These figures paint a rather similar picture: The majority of people strongly agreed that the VR application is more fun and more motivating to use and no participant strongly disagreed. However, this agreement in the “fun”-question was expressed by more people (19 vs. 13) than in the “motivating”-question with more people choosing the less decided answers of 2-4 when it comes to motivation.

Regardless of this difference, the overall tendency shows that fun and motivation are more pronounced in VR than in the web application. This can be due to VR being more unfamiliar to most users than a web application and therefore being more interesting. Yet this data still shows that using the VR app seemed to be a positive experience for most users.

Both the fun and motivation expressed by participants negatively correlated with the general UML experience of participants. According to Spearman’s rank correlation, both had a significance level of < .05, while the *fun*-related answers showed a correlation coefficient of -.433 and the *motivation* ones -.431. This means that the participants more experienced with UML found the difference in fun and motivation between the VR and the web applications less pronounced.

**Fig. 23:**
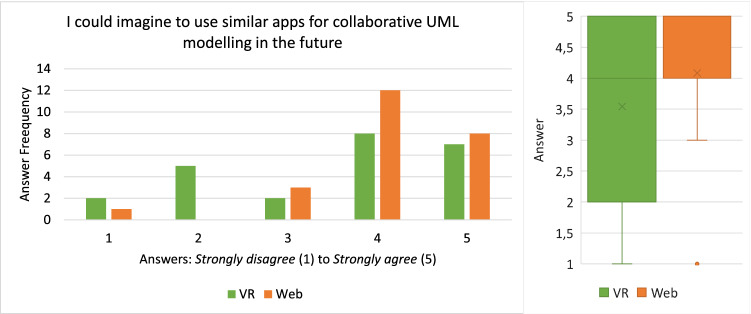
Participant’s intention to use the tools for collaborative modeling

*Intention to use the tools in the future* As a resumé, the questionnaire also included two questions regarding the intention of the user to use applications like the web and VR ones in the future. Figure 23 visualizes the results of these questions side by side.

These charts show that 20 out of 24 people would like to use the web application in the future while only 15 answered similarly for the VR app (answers 4 & 5). Accordingly, seven people stated they would not want to use an app like the VR one, while only 1 person answered like that for the web application (answers 1 &2).

This difference can be observed again when looking at the mean values of 4.1 for web and 3.5 for VR. According to a Wilcoxon signed-rank test, this difference was not statistically significant, however, producing a $Z = -1.498$ and a significance level > .05.

An analysis with Spearman’s rank-correlation showed that the intention to use web applications in the future correlates with both the general UML experience and the experience with UML tools of the participants. Both showed a significance of < .05 with general UML experience having a correlation coefficient of .428 and UML tool experience a .419.

**Fig. 24:**
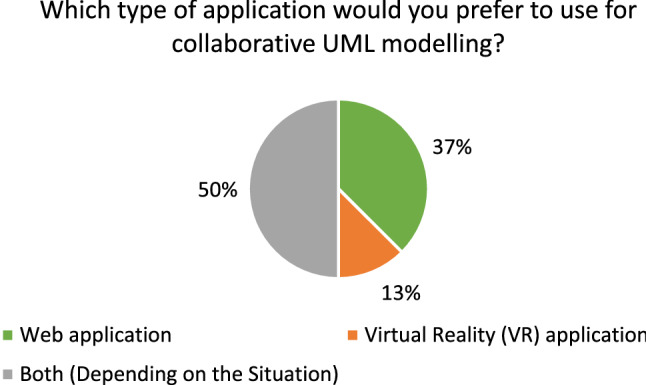
Participant’s preference after using the applications

*Application preference* In one of the last questions, participants had to choose which application they would prefer to use for collaborative UML modeling: They could choose either one or state that they would want to use both depending on the situation. A follow-up question to this one subsequently asked in which situations they would want to use which application if they selected “Both.” Figure 24 shows the preferences as indicated by participants.

13% (3 out of 24) wanted to use the VR app over the web application and 37% (9 out of 24) vice versa. Half of the participants, however, indicated that they would want to use both applications, each for specific situations.

When asked in which situations they would want to use which application, participants gave rather diverse answers. Five users stated that they would want to use the web application for complex or longer tasks and the VR one for shorter ones. Two mentioned that they would prefer the web application for time-critical work. Brainstorming and planning was mentioned by three people to be more suited for the VR application and one person stated that she would like to use “the web application when you are working on one device with your partner” and the VR app when in “different locations.”

*General remarks and feedback* Apart from the above-mentioned questions, we asked the participants open-ended questions to provide us general feedback and remarks (what have you liked/disliked most) on the developed collaborative VR modeling environment. The most notable result is that 14 out of 24 people mentioned that moving the model elements through the grabbing feature felt natural. Furthermore, most comments were praising the “collaboration” in the VR modeling environment. Some comments explicitly mentioned a like for the collaboration (3 out of 24), some indicated a positive impression for the support to talk to the teammate (4 out of 24), being able to see the teammate (4 out of 24) or the feeling of the teammate’s presence (4 out of 24). Some participants mentioned multiple of these aspects. In total, 11 out of 24 noted that at least one of these collaboration-related aspects felt natural or intuitive. Concerning the negative feedback comments, typing on the keyboard in VR was the most mentioned aspect (9 out of 24), while some of the participants stated that this would only need some time to get used to it. Furthermore, the missing of an auto-alignment or snapping feature, like known from most diagramming and modeling tools (e.g., Microsoft Visio [30]), was complained about by five users. However, five out of 24 people said that VR was entertaining or fun to use and two added that it is specifically useful for home office scenarios since it makes people feel more together and would “definitely improve motivation and team spirit.”

#### Discussion

Based on previous research and the participants’ familiarity with traditional computer programs, we expected one downside of VR to be that users’ task executions are slower and more error-prone in VR. The data from our study show that this was indeed the case. These measures could have been influenced by the universal familiarity of participants with PC applications in general and UML tools specifically. Therefore, it is possible that speed and error rates in VR improve as users get more experience with a specific VR UML tool. However, the current state-of-art text entry can be seen as a major bottleneck for the efficiency of text-intensive VR applications. The results of the SUS evaluation additionally showed that our VR implementation is already quite usable even though it still lacks many features that users expect from such an application like automatic aligning of model elements and copy & paste functionalities. The various data points gathered about the naturalness of different aspects of the application gave a clearer insight on what concrete advantages such a VR application can have compared to traditional tools: On average, users found the interactions and especially the collaboration related aspects of the VR application significantly more natural than in the web application. This is especially important with respect to this work’s focus on remote collaboration settings as the study showed that the feelings of being together and collaborating face to face with a co-worker were much higher in VR compared to the traditional PC alternative. Another aspect that we expected VR to be beneficial for is the motivation and fun users are having while using it. Our study shows that it is indeed true that users were a lot more motivated and had a lot more fun using the VR application compared to the web app. It is important to note that this could be influenced by the fact that VR is a relatively new and therefore possibly more interesting technology, so these values might align more over time when a user regularly uses a VR application for modeling. The fact that using VR can be more exhausting for people was one downside of VR we expected to observe in this study as well. While we did see that some participants experienced significant discomfort (due to heavy headsets, pressure against the face, or cybersickness), most people did not have any issues with that.

Considering the suggestions made by participants about when they would like to use which application, it is evident, however, that a VR UML tool would not simply replace the desktop ones. It would rather be an amendment, so users have the option to use the tool that is most suited for a given situation. In remote collaboration settings, where two or more people need to brainstorm or discuss how something should be modeled, the VR application could be used. When collaborating in the same room or when creating a model alone, a PC-based application could be more appropriate. This implies that such apps need seamless interoperability between PCs and VR when they ought to be used in actual modeling work outside of usability studies. To summarize, it can be said that VR can offer a more natural collaborative modeling experience compared to PC-based tools but that both techniques have certain advantages and drawbacks. These make the use of both tool-types, depending on the concrete situation, most sensible instead of using only one of them exclusively.

#### Threats to validity

With regard to collaboration, this study only evaluated two-person teams. Therefore, it is unclear if the findings can be generalized to larger numbers of simultaneous collaborators. The number of participants and thereby collaboration teams was also rather limited. With more participants, a between-subjects design [3] could have been chosen which has the possibility to provide data and thus findings that are more generalizable across many different users. Due to the participants’ demographics (e.g., age, profession, background, etc.), the findings of this study should be taken carefully and further user studies with other user groups are needed to get insights about the general usability. Additionally, this study compared a fully featured commercially available web application with a VR modeling prototype, missing many of the features that the web application supported, like automatic aligning of model elements. This study therefore could not achieve a strict like-for-like comparison between the two types of applications.

## Conclusion and outlook

While the Unified Modeling Language (UML) is one of the major conceptual modeling languages for software engineers, more and more concerns arise from the modeling quality of UML and its tool-support. Among them, the limitation of the two-dimensional presentation of its notations and lack of natural collaborative modeling tools are reported to be significant. In this paper, we have explored the potential of using virtual reality (VR) technology for collaborative UML software design by comparing it with classical collaborative software design using conventional devices (desktop PC/laptop). For this purpose, we have presented a VR modeling environment that offers a natural collaborative modeling experience for UML Class Diagrams. Based on a user study with 24 participants, we have compared collaborative VR modeling with conventional modeling with regard to efficiency, effectiveness, and user satisfaction. The main results show that the use of VR has some disadvantages concerning efficiency and effectiveness, but the user’s fun, the feeling of being in the same room with a remote collaborator, and the naturalness of collaboration were increased.

In ongoing work, we are developing a gamification-based UML learning environment in VR [59] that is based on the approach presented here. The VR environment provides minigames and multi-viewpoint modeling features to learn creating class diagrams. Furthermore, this environment supports a multi-modal interaction and a more realistic representation of the virtual characters. Besides these improvements, we further plan to implement features like automatic alignment of model elements, and tracking of which collaborator created which parts of the model. Apart from that, scalability and performance are important technical aspects that should be investigated in future work, especially if larger groups of people use such a VR modeling tool for collaboration purposes. In addition, further usability evaluation studies with larger groups of heterogeneous participants and more complex modeling tasks should be conducted to analyze in more detail the benefit of collaborative modeling in VR. Finally, we believe that a cross-device mixed reality collaborative modeling approach is a promising way to support modeling across different AR/VR capable and conventional devices.

## Footnotes

1 https://secondlife.com/.

2 https://www.oculus.com/quest-2.

3 https://www.oculus.com/rift-s.

4 https://github.com/Blanner/VmodlR.
